# Development of Lightweight Building Materials Using a Sustainable Chemistry Approach: The Multifunctional Effects of Garlic Husk Ash on Foam Concrete

**DOI:** 10.1021/acsomega.5c06754

**Published:** 2025-11-22

**Authors:** Mehmet Uğur Yılmazoğlu, Halil Oğuzhan Kara, Kenan Toklu, İffet Gamze Mütevelli Özkan, Ihsan Türkel, Mahmut Bilgehan, Adem Ahıskalı, Oğuzhan Yavuz Bayraktar, Gökhan Kaplan

**Affiliations:** † Department of Civil Engineering, 187466 Kastamonu University, Kastamonu 37150, Turkey; ‡ Department of Civil Engineering, Tekirdağ Namık Kemal University, Tekirdağ 59850, Turkey; § Department of Civil Engineering, Atatürk University, Erzurum 25030, Turkey

## Abstract

In this study, the application of garlic husk ash (GHA) as a new form of biomass-related pozzolanic material that could be used as a partial replacement of cement in the production of foam concrete was examined. GHAs added to cement were equal to 0–30 wt %. The impact of GHA on various properties tested (i.e., mechanical strength, durability, thermal conductivity, and microstructure) demonstrated that GHA is a silica, low-calcium material. Adding GHA increased the initial and final set times by up to 56 and 52%, respectively. As water demand increased, workability declined. Despite the decrease in early age compressive strength, later-age strength improved due to pozzolanic activity. At 90 days, the GHA30 mix’s compressive strength reached 12.80 MPa, slightly exceeding that of the control mix. Insulation properties improved as thermal conductivity decreased by 18% to 0.199 W/m·K. After 50 freeze–thaw cycles, the GHA25 and GHA30 mixes maintained up to 87% of their compressive strength, demonstrating exceptional freeze–thaw resistance. In high-GHA specimens, SEM analysis confirmed the development of a denser microstructure with increased C–S–H gel. The outcomes indicate that GHA, as an abundant biomass byproduct, enhances the long-term durability of lightweight concrete while minimizing the environmental footprint associated with cement utilization. This research promotes the integration of agricultural biomass residues into construction materials to improve circular economy principles and foster the development of sustainable, energy-efficient infrastructures.

## Graphical abstract

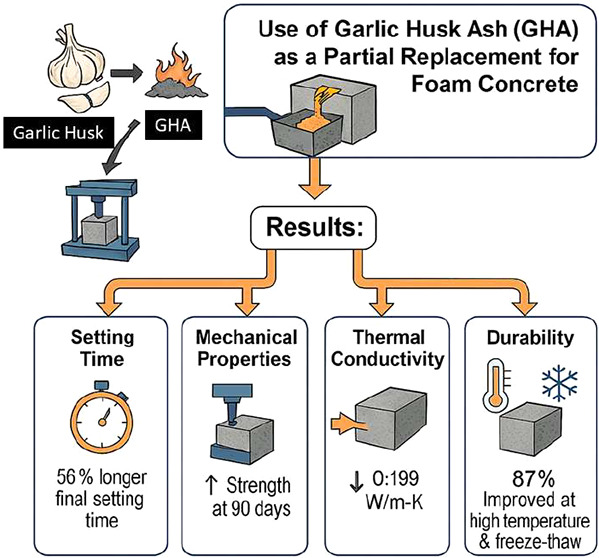

## 1 Introduction

One of the great quests of this century is the search for sustainable means in engineering practice based on the Sustainable Development Goals (SDGs) formulated by the United Nations and advised by us.^1,2^ From the UN’s recommended Sustainable Development Goals (SDGs) to incorporate the circular economy into waste management, massive urbanization and population growth in metropolitan areas have created a conundrum of cleaner production and consumption to address insurmountable urbanization problems, such as waste generation.^3^ This massive increase in waste production is a significant obstacle to sustainable development, as is the increased CO_2_ production in recent years. This is why new approaches to waste management, such as the circular economy, which considers each material’s life cycle, are increasingly being adopted in different sectors, such as construction.^1^ There is a global recognition that the policy of producing, using, and discarding is unsustainable and has negative environmental, economic, and public health impacts. In recognition of these concerns, governments worldwide are looking for scientific solutions to recycle all waste materials to develop a closed-loop circular economy where today’s waste is tomorrow’s raw material.^4,5^

It is consistently reported that the construction sector uses almost 40% of total energy production and that cement production alone accounts for 8% of total global CO_2_ production.^6^ In this sector, the inexorable demand for building materials and binders has driven ongoing research into the most efficient means of producing a more sustainable binder, particularly by exploring new systems that make greater use of waste materials. In this context, blended cements^7,8^ and Supplementary Cementitious Materials (SCM)^9,10^ have gained importance in the production of traditional and special concretes. Industrial, agricultural, and municipal wastes are primarily used to make such composites because of the importance of sustainability, such as SCM. Especially in recent years, the rate of R&D studies on using agricultural wastes such as rice husk ash (RHA), palm oil fuel ash (POFA), and corn cob ash (CCA) has increased. In addition, new studies on alternative agricultural waste continue to be conducted. Garlic husk ash is one of these alternative SCMs derived from agricultural waste.

Although they have interesting properties, garlic waste materials such as peels, husks, stems, and straws have no industrial use.^11^ Solid waste, such as garlic husks and straw, accounts for about 30% of the garlic components. Therefore, garlic wastes generate significant solid agricultural waste every year.^12^ Garlic (Allium sativum) is used in many parts of the world, including Asia, North Africa, Central America, and the Middle East, and it is part of the same family as onions. The annual world production of garlic (Allium sativum) is around 20 million tons. China and India are the biggest producers of garlic. 2018–2019, India alone produced about 1.1 million tons of garlic.^13^ In Turkey, at least 100,000 tons of garlic have been produced annually since 2016. This value increases every year. Today, garlic production is thought to be approaching 150,000 tons. Approximately 25% of garlic in Turkey is produced by agricultural land in Kastamonu province.^14^ Garlic corms produce roughly 760 g/kg cloves and 240 g/kg outer and inner husks.^15^ These waste husks, which are released at approximately 25% during garlic production, will constitute a significant amount worldwide.

Using agricultural waste in building materials has become essential to sustainable construction practices. In particular, wastes with high silica and pozzolanic content have the potential to reduce environmental impacts by partially or fully substituting conventional cement. Agricultural wastes are generally calcined in cement-based composites and used in ash form. In this way, it will be possible to use garlic husks in cement-based composites. In this context, garlic husk ash (GHA), a recyclable regional waste, offers an important alternative that can be utilized as a binder or additive material in building materials. The potential of such agricultural waste is particularly relevant in the production of foam concrete, where criteria such as lightweight properties, insulation, and environmental sustainability are prioritized. However, there is no research on using garlic husks, a regional waste, to improve the properties of lightweight cement-based composites (such as foam concrete).

Foam concrete is lightweight, porous concrete enriched with random air holes. It can be classified as lightweight concrete with a 400 kg/m^3^ density to 1850 kg/m^3^.^16,17^ Foam concrete has many advantages over ordinary concrete, such as lightweight, sound insulation, heat insulation, low elasticity and seismic resistance, durability, fire resistance, fluidity, and easy plasticity. It is also environmentally friendly.^18–20^ Also, foam concretes are widely used in constructing lightweight buildings, road sub-bases and house partitions because they are easy and inexpensive to produce and apply.^21,22^ Today, it is recognized as a win-win strategy for sustainable waste management and the greening of foam concrete, incorporating recycled solid waste and industrial and agricultural byproducts to produce new foam concrete.^23^ As a result, researchers and academics have carried out several studies on foamed concrete to dispose of solid waste.^24–28^ In this context, more and more studies have been conducted in recent years to improve, develop, and modify the properties of foam concrete made with agricultural wastes, which now constitute an essential part of solid waste. In studies conducted by Mydin et al., it was shown that up to 20% RHA increased compressive strength and reduced porosity.^29^ POFA from the palm oil industry has become an adequate partial replacement for cement, exhibiting pozzolanic properties. Studies show that using 10% POFA can optimize the compressive strength, while up to 20% can match the strength of conventional foamed concrete.^30^ Cottonseed protein acts as a foaming agent. It has been determined that it can be combined with solid waste to form foam concrete with favorable properties, including high compressive strength and thermal resistance.^31^ Alnahhal et al. used POFA as cement replacement material at 10, 20, and 30% and achieved a target oven dry density of 1300 kg/m^3^. Their study showed that POFA could be an alternative replacement material in foam concrete production.^32^ Salari et al. investigate the performance of palm oil clinker (POC) as a replacement for cement and sand in foamed concrete. The study explores the compressive, flexural, tensile strengths, and elastic modulus of lightweight foam concrete (LFC) containing POC and thermally activated POCP (TPOCP). The results show the potential of POC and TPOCP to improve the strength and durability of LFC.^33^ Reddy and Vinod obtained high strength and durability in lightweight foam concrete containing CCA and basalt fiber (BF). They recommended a foam content of 50 kg/m^3^ in a mix containing 50% CCA and 1.5% BF as the optimum mix ratio.^34^ Mohamad et al. produced foam concrete with banana skin powder (BFS) and POFA. The increase in the percentage of BSP and POFA included in foam concrete showed a slight increase in mechanical properties.^35^ Zhao et al. obtained very low thermal conductivity coefficients (generally less than 0.06 W/m·K) in foam concretes produced with agroforestry wastes (such as RHA and plant ash).^36^ In the study by Jhatial et al., eggshell powder (ESP) at 5–10% increased the compressive and tensile strength of foam concrete due to its high calcium content, and, when combined with POFA, significantly improved microstructural density and durability by promoting secondary C–S–H formation. These results indicate that calcium-rich agricultural wastes can be an effective mineral admixture in sustainable binder systems.^37^ Many studies also show that agrarian waste ash improves foam concrete properties.^38–40^ However, there are no studies on using ashes from garlic production waste in foam concrete or cement-based composites.

Agricultural waste-derived pozzolanic materials such as RHA, SCBA, and POFA have been extensively researched in the literature. These materials contribute to strength development mainly due to their high amorphous silica content. GHA, however, stands out from these materials in that it has a high loss-on-ignition value and, with its porous structure, significantly reduces thermal conductivity in foamed concrete. This study experimentally evaluates GHA as a binder admixture in foam concrete production for the first time in the literature. The contribution of this regional waste from agricultural production to building materials with multiple performance criteria, such as lightweight, thermal insulation, and sustainability, is discussed within the framework of circular economy principles. Although some agricultural wastes such as RHA, POFA, and ESP have been shown to improve foam concrete properties in the existing literature, the use of GHA in this context has never been investigated. In this context, the originality of this study lies in its ability to fill a gap in the literature and, for the first time, reveal the effects of GHA, a pozzolanic admixture with high calcium and silica content, on the mechanical and microstructural properties of foam concrete. In addition, the study makes essential contributions to environmental sustainability, resource efficiency, and waste management by enabling the utilization of local resources in building material production through the recycling of regional agricultural waste.

## 2 Materials and Methods

### 2.1 Raw Materials

In this study, CEM II A-LL 42.5N type cement was preferred because it provides both physical and chemical filling effects during the hydration process, thanks to the limestone additive in its content; thus, it supports the matrix to gain a more compact and homogeneous structure, especially in foam concrete systems with a porous structure. The literature states that CEM II cements provide more stable setting times and controlled heat release in systems with low density and high void volume, such as foam concrete. In addition, since CEM II cements have a lower clinker ratio than CEM I cements, they are more environmentally friendly regarding CO_2_ emissions from production. In this respect, it also aligns with the study’s sustainability-oriented approach. The specific gravity of CEM II A-LL 42.5N cement is 2.93, and the specific surface area (Blaine method) is 4200 cm^2^/g. GHA was replaced by cement in the production of foam concretes. GHA was obtained from the waste site of the garlic processing plant in Kastamonu province. The specific gravity of GHA is 2.27, and the specific surface area (Blaine method) is 3300 cm^2^/g. GHA was obtained from the landfill of garlic production facilities. The inner and outer husks from garlic bulbs were burned with the help of a stove (uncontrolled burning). Uncontrolled burning was chosen because it better reflects the reality (rural conditions) due to its ease of implementation and low cost (Figure 1). A cycle of 24 h was determined for the burning and cooling process. The GHA obtained from combustion was ground into powder form by a ball mill for 1 h. All particles of GHA passed through a 125 μm sieve. The chemical compositions of cement and GHA are presented in Table 1. Mineralogical and particle size distribution of GHA are given in Figure 2.

**Figure 1:**
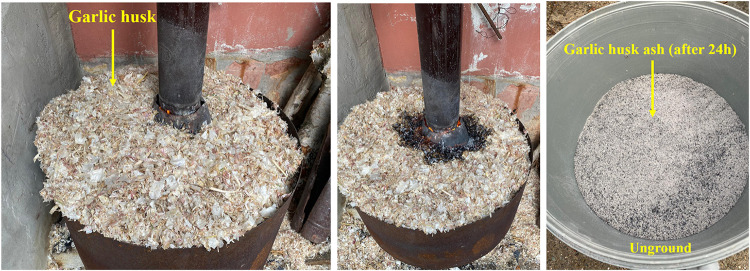
Stages of obtaining GHA^41^ (Yılmazoğlu, 2025) under the creative commons license CC BY 4.0.

**Figure 2:**
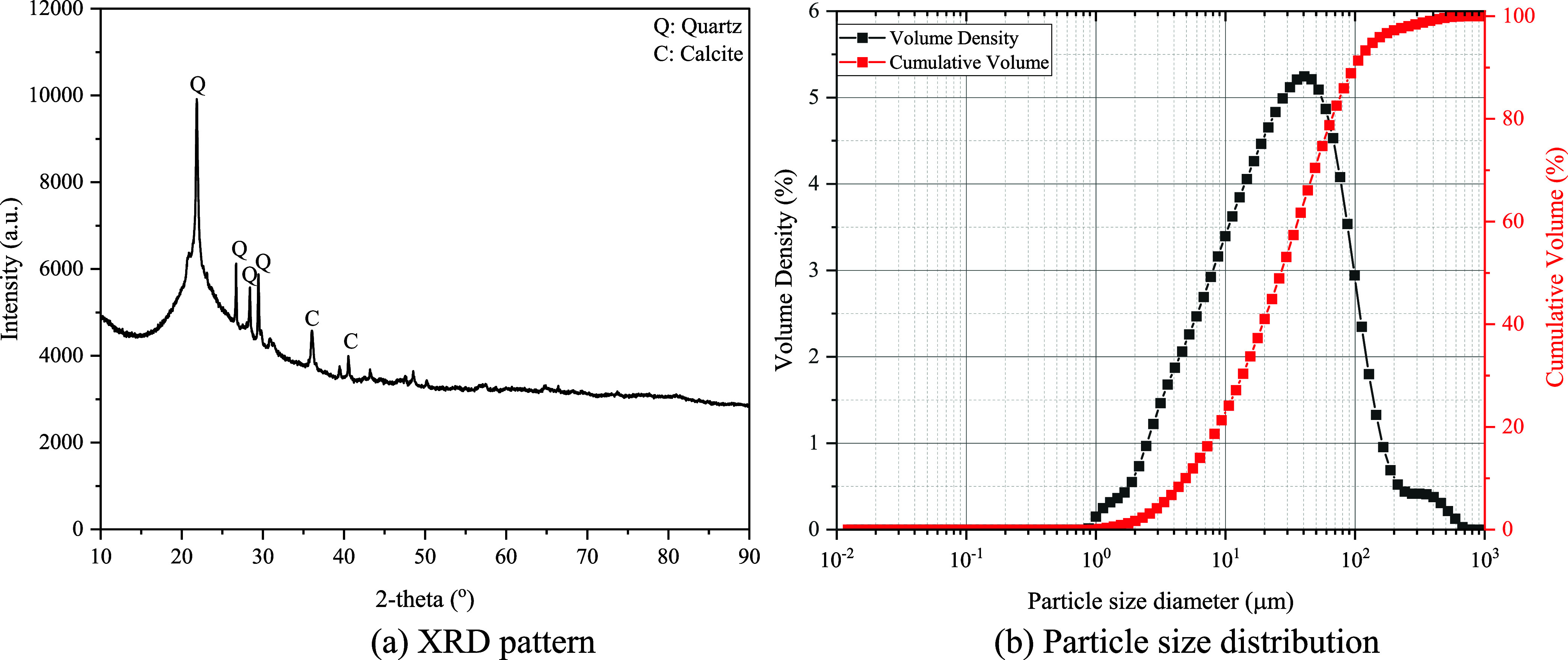
Mineralogical and physical properties of GHA.

**Table 1:** Chemical Compositions of Cement and GHA

Oxide (%-by weight)	CaO	SiO_2_	Al_2_O_3_	Fe_2_O_3_	SO_3_	MgO	Na_2_O_(eq)_	LOI^a^
CEM II A-LL 42.5N	60.5	20.7	5.9	3.6	3.7	2.3	0.6	2.6
GHA	26.3	25.8	3.7	4.7	3.5	2.4	5.9	27.6

a Loss on ignition.

The SEM image shows that GHA particles have a highly irregular, uneven, and porous surface morphology (Figure 3). This irregular morphology can improve the cement paste’s interfacial transition zone (ITZ) by providing better mechanical interlocking with the binder matrix. Furthermore, GHA can act as a nucleation center for hydration products due to its high specific surface area. In the other image (bottom side), more significant, sharp-edged, irregular particles and some glass-like structures are more clearly noticeable. This morphology indicates that GHA may have pozzolanic activity regarding its glassy phase content. Sharp-edged particles can create a microfilling effect in the cement matrix and reduce workability by increasing water demand. In addition, it was determined in SEM images that it is an industrial waste with a hollow structure and high porosity. The ash obtained after combustion was gray in color, but after grinding, the ash turned dark black due to the smaller particle size and more visible carbon residues on the surface (Figures 1 and 3).

**Figure 3:**
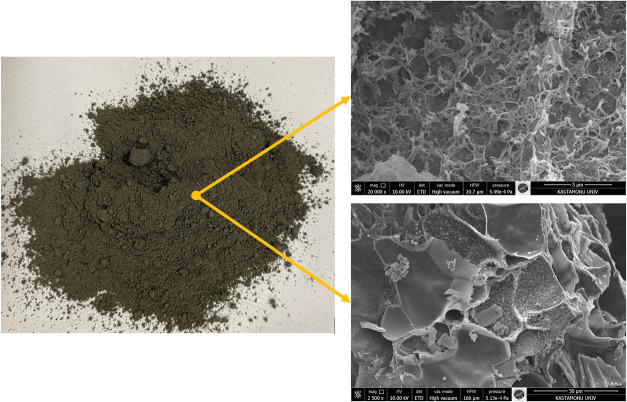
Physical appearance and microstructure of GHA^41^ (Yılmazoğlu, 2025) under the creative commons license CC BY 4.0.

The most remarkable region in the XRD pattern (Figure 2a) is a very prominent and broad peak in the 20–30° (2-theta) range. This indicates an amorphous phase, i.e., GHA contains an amorphous, glassy structure. Amorphous silica (SiO_2_) is more prone to pozzolanic reactions, indicating binding potential. This pattern suggests that GHA contains reactive amorphous silica and a certain proportion of crystalline inert minerals. GHA exhibits a fine-grained ash structure with a large surface area (Figure 2b). The median size (d50) is around 26 μm, indicating that it is in the particle size range suitable for pozzolanic reactions. The symmetrical structure of the curve shows that the particle sizes are not spread over an extensive range, i.e., the distribution is relatively narrow and homogeneous. This will provide better packing and reactivity in the mixture.

Pumice aggregate with a sieve opening of 0–4 mm produced foam concretes with and without GHA content. The particle size distribution of pumice aggregate according to ASTM C33 standard is presented in Figure 4. The specific gravity of pumice aggregate is 1.82, and water absorption (24 h) is 10.5%. This study used a commercially available protein-based foaming agent to produce foam concrete. The foaming agent was made by mixing with water in a particular ratio (1:50) using a high-speed mixer (>3000 rpm). The agent used has a surfactant content that is environmentally low in toxicity, biodegradable, and compatible with cement. The prepared foam was incorporated into the mix to provide a volume-stabilized cellular structure. The density of the foam produced was approximately 120 kg/m^3^, and the foam volume was kept constant. The foaming agent used improved the workability of the mixture and significantly affected the thermal insulation capacity and lightweight properties of the postcured structure. The stability of the foam and its compatibility with the matrix contributed positively to the final structural performance by ensuring the homogeneous distribution of the cells. Potable municipal water was used to prepare the mixtures (for workability and hydration) and during curing.

**Figure 4:**
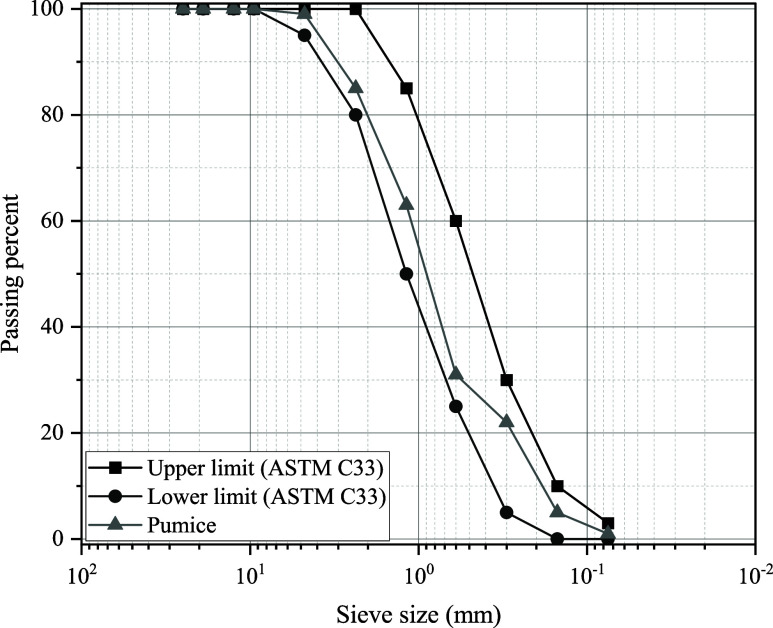
Particle size distribution of pumice aggregate.

### 2.2 Mix Design

All foam concrete mixes produced in this study were designed by maintaining a constant water/binder ratio and foam volume. CEM II A-LL 42.5N type cement and different proportions of GHA were used as binder phases in the mixtures. Seven different mix groups were formed by substituting GHA for cement at ratios of 0, 5, 10, 15, 20, 25, and 30 wt % of the binder phase. These substitution ratios were named as GHA0, GHA5, GHA10, GHA15, GHA20, GHA25, and GHA30, respectively. Thus, the effects of GHA on the performance of foam concrete were systematically evaluated. The total amount of binder was kept constant in all mixes, with only a gradual decrease in cement due to GHA substitution. The reference mix with pure cement (GHA0) contained 500 kg/m^3^ CEM II A-LL 42.5N, while in the GHA30 mix, this ratio was adjusted to 350 kg/m^3^ cement + 150 kg/m^3^ GHA. The amount of mixing water used in each mix was fixed and determined as 250 kg/m^3^. The water/binder ratio (w/b) was designed to be 0.5 for all mixtures, and the share of GHA in the binder phase was included in this ratio.

To form the cellular structure of the foam concrete, a fixed amount (50 kg/m^3^) of protein-based foam was preproduced and included in all mixes at the same volume ratio. In this way, the mixtures’ variability in density and porosity comparisons was minimized. In addition, natural pumice stone with a grain size of 0–4 mm was used as aggregate in all mixtures. As the binder ratio decreased depending on the amount of GHA, the aggregate amount gradually reduced at a small rate (for example, 320 kg/m^3^ in GHA0, 297 kg/m^3^ in GHA30). In this way, the total solid content was stabilized, and the consistency of the mixture was maintained. The material quantities of the mixtures with and without GHA are given in Table 2.

**Table 2:** Material Quantities of Mixtures with and without GHA

kg/m^3^	GHA0	GHA5	GHA10	GHA15	GHA20	GHA25	GHA30
CEM II A-LL 42.5N	500	475	450	425	400	375	350
GHA	0	25	50	75	100	125	150
Water	250	250	250	250	250	250	250
Foam	50	50	50	50	50	50	50
Pumice	320	316	313	309	305	301	297

### 2.3 Mixing, Casting and Curing

All foam concrete mixes were prepared using a controlled three-stage mixing procedure to ensure a homogeneous matrix and stable cellular structure. In the first stage, binder constituents (i.e., CEM II A-LL cement and GHA) and pumice aggregate were dry mixed for 1 min in a low-speed (150 rpm) laboratory-type mechanical mixer. This mixing was critical to ensuring a homogeneous distribution of the binder with the aggregate. In the second stage, the defined quantity of mixing water was added slowly, and mixing proceeded at medium speed (300 rpm) for another 2 min. This stage is crucial for establishing the desired consistency and allowing the binders to react. In the third stage, foam (density approximately 120 ± 5 kg/m^3^), which was previously prepared with water and foaming agent, was added to the mixture. The foam was fed into the mix at low speed (150 rpm) for 2 min to ensure a homogeneous distribution of air bubbles into the matrix without deflating. All mixtures were prepared following the same mixing time and sequence, thus minimizing production-related differences between different mixtures.

The fresh mixture was placed in cube molds measuring 50 × 50 × 50 mm and prism molds measuring 40 × 40 × 160 mm, and light vibration (with a mallet) was applied to each mold during casting to ensure homogeneous distribution of the foam. The specimens were kept in the molds for 24 h in the laboratory environment (23 ± 2 °C, relative humidity 50 ± 5%) and then removed from the molds. The specimens were kept in lime-saturated water until the test day to ensure the development of postsetting properties. These curing conditions are crucial for completing the binding reactions (hydraulic and pozzolanic) and developing strength-gaining products within the structure without compromising the internal moisture of the foam concrete. All physical and microstructural tests were carried out after 28 days of curing.

### 2.4 Test Method

This study conducted extensive experimental tests to determine the fresh, hardened, physical, mechanical, and durability properties of foam concrete mixtures. Internationally recognized standards were followed in all experiments. The tests were carried out in two stages: paste and mortar tests. Since the properties of GHA-containing cement-based composites have not been previously investigated in the literature, paste tests were carried out first. The binder phase’s initial and final setting times were determined with a standard Vicat instrument following the TS EN 196-3 standard. Within the scope of the same method, the amount of water of consistency was also determined for each mixture. Expansion (soundness) values were obtained using the Le Chatelier apparatus under the TS EN 196-3 procedure. A mini flow table determined the workability properties of foam concrete according to ASTM C1437 standard, and flow diameters were measured in millimeters. Fresh density measurements were made using the TS EN 12350-6 standard by taking the volumetric mass of the fresh mixture.

Apparent porosity was determined by the volumetric method and calculated by measuring dry and saturated weights after the samples were saturated. The oven dry density measurements were made by drying the samples at 50 ± 5 °C until reaching constant mass and evaluated according to the TS EN 12390-7 standard. In this context, 50 × 50 × 50 mm cube samples were produced. Physical properties were determined at the end of the 28-day curing period. Immersed and capillary absorption methods were used to investigate the water absorption behavior of the specimens. Both tests were conducted on 28-day samples based on TS EN 772-11 and TS EN 1015-18 methods. The amount of water absorption was calculated from the mass differences of the samples before and after immersion. Cube specimens with a cross-section of 50 × 50 × 50 mm were produced for these tests. Water absorption tests were conducted after 24 h as specified in EN 772-11 and EN 1015-18 standards, and the final values at these times were reported instead of the full data series.

Compressive and flexural strength tests were conducted on 40 × 40 × 160 mm prism specimens aged 3, 7, 28, and 90 days following the TS EN 196-1 standard. The three-point bending method was used to perform Flexural strength measurements on 40 × 40 × 160 mm prisms. At least three specimens were evaluated for each test group, and average results were reported. A 2000 kN capacity hydraulic test press was used for compressive strength testing. A 50 kN capacity testing machine was used for flexural strength testing. Both machines were calibrated annually with reference load cells.

Volumetric changes due to drying were measured on 25 × 25 × 285 mm mortar bars per the ASTM C596 standards. After 7 days of water curing, the initial lengths of the specimens were determined, and drying shrinkage was measured from this day until the 120th day. The samples were kept in laboratory conditions (23 ± 2 °C temperature and 50 ± 5% relative humidity) until the 120th day. The length changes were measured at some intervals using a digital comparator.

Freeze–thaw (F-T) resistance testing was conducted to establish the resistance of foam concrete samples to environmental adversity. The test procedure was based on ASTM C666 (Procedure A) standard. For F-T cycles, an automatic programmable device was used between −20 and +20 °C. Calibration of the device was done with thermocouples. Each mix’s 40 × 40 × 40 × 160 mm prismatic specimens were subjected to freeze–thaw cycles after 28 days of water curing. In the experimental program, the specimens were frozen at −18 °C for 4 h and thawed at +20 °C for 4 h. A total of 50 cycles was carried out. During the freeze–thaw cycles, samples were kept saturated, and excess water was carefully removed from the surface between each cycle. After the tests, the compressive strength and weight loss from three samples for each mix were recorded, and the average values were taken. The weight loss was calculated by expressing the difference between the sample weights before and after the cycle as a percentage. In addition, the strength loss rate was evaluated by comparing it with the reference compressive strengths measured after 28 days of curing.

Thermal strength performance was investigated on a 28-day specimen furnace-fired at 250, 500, and 750 °C. The specimens were kept at the specified temperatures for 2 h and then brought to room temperature, and both mass loss and residual compressive strength were measured. ASTM E119 and high-temperature behavior analyses in the literature were taken as references for these tests. 50 × 50 × 50 mm cube specimens were used for these tests. In addition, the heating rate of the muffle furnace used for the high-temperature effect was 10 °C/min. The thermal conductivity coefficients of the specimens were determined on 28-day oven-dried plaque specimens (300 × 300 × 30 mm). Thermal conductivity coefficients of foam concrete were determined following the ASTM C518 standard. Thermal conductivity was determined using a heat flow meter. The device was calibrated using an EPS panel with a known thermal conductivity coefficient. The measuring range of the device is 0.02–2.0 W/mK. The equipment and devices used in the experimental study are given in Figure 5.

**Figure 5:**
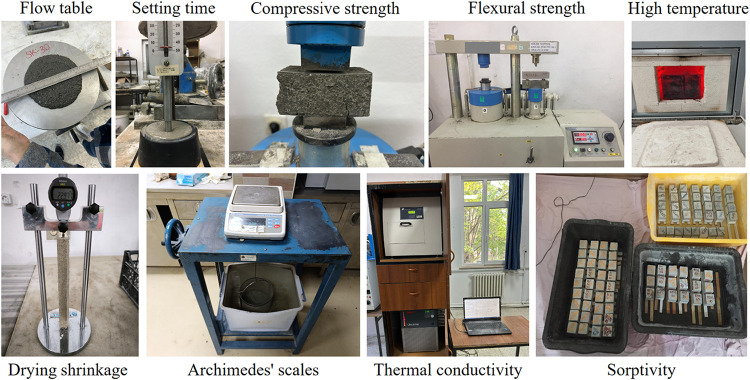
Equipment used in the experimental study.

Microstructural analyses were performed on the selected samples using scanning electron microscopy (SEM). These analyses evaluated pore structure, C–S–H formation, distribution of GHA in the matrix, and void ratios. The samples were made conductive with gold plating and examined under 250–10,000× magnification under a microscope. SEM examinations were carried out with the FEI brand’s Quanta FEG 250 model device at the MERLAB unit of Kastamonu University. XRD analyses were performed on a Bruker D8 Advance model instrument. The scanning range was between 10 and 90°, and Cu–Kα (λ = 1.5406 Å) was used as the radiation source. The flow diagram of the experimental study is presented in Figure 6.

**Figure 6:**
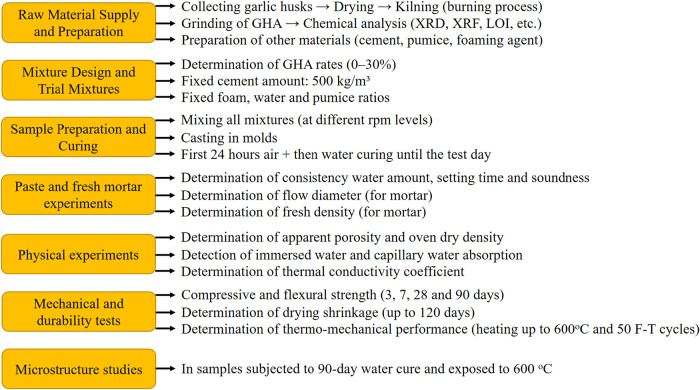
Flowchart of the experimental study.

## 3 Results and Discussion

### 3.1 Paste Experiments

The setting times of the pastes with and without GHA content are given in Figure 7a. The initial setting time for the reference mixture GHA0 is 115 min. With a 10% substitution of GHA, this time increases to 135 min, corresponding to an extension of approximately 17.4%. In the mixture with the highest substitution rate of 30% GHA, the initial setting time reached 180 min. This shows a 56.5% extension of the setting time compared to the reference mix. This is primarily because GHA has a lower hydration capacity and soluble calcium content than cement. According to the chemical composition table, CEM II A-LL cement contains 60.5% CaO, whereas this ratio is 26.3% in GHA. This difference caused a delay in the formation of calcium silicate hydrate (C–S–H) during the hydration process. At the same time, the high silica (SiO_2_) content of GHA (25.8%) causes the pozzolanic reactions to start late because these reactions develop over time with the hydration products of the cement. According to several studies, the rice husk ash (RHA), coconut ash, and other ashes from agricultural wastes increase the setting time in the cement matrix. For instance, as Ganesan et al. (Ganesan et al., 2008) reported, the setting time has increased by 20–50% when RHA is substituted and this was attributed to the secondary reaction of the mechanism.

**Figure 7:**
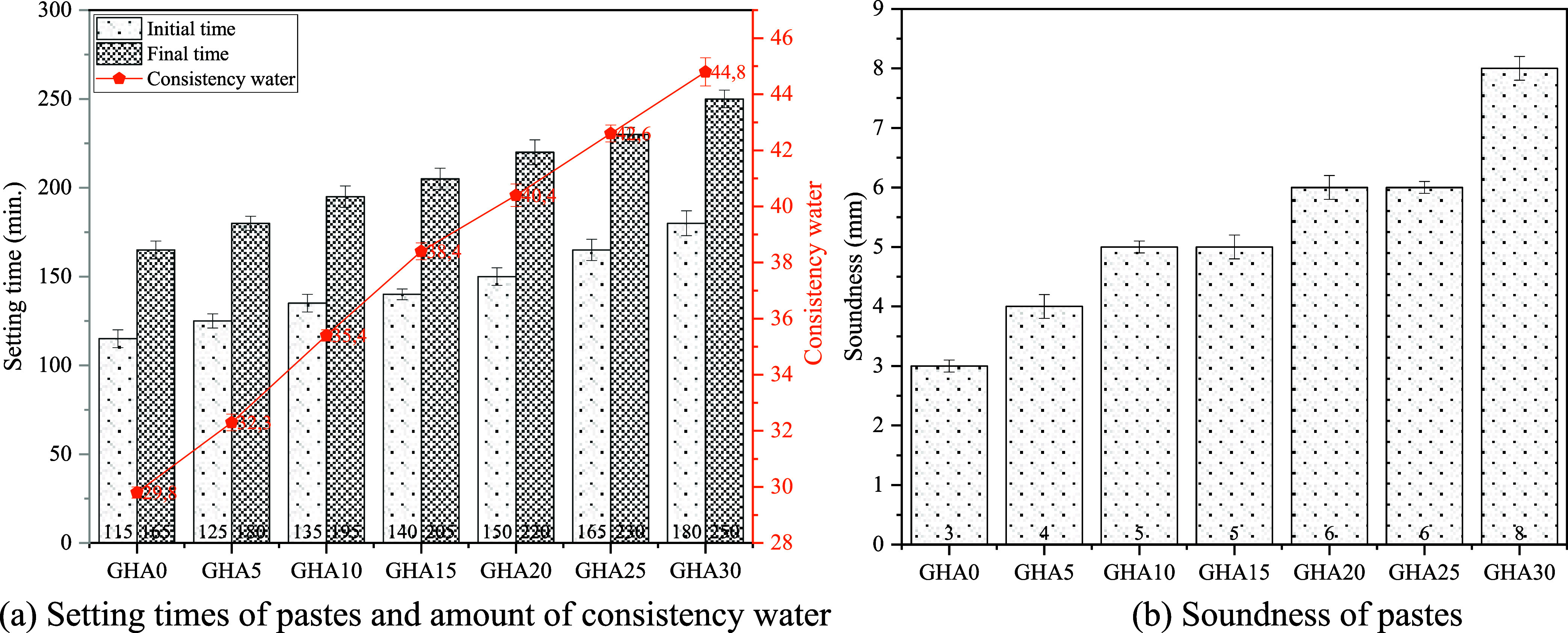
Physical properties of pastes.

The final setting time, which was 165 min in the GHA0 mix, reached 195 min when GHA was 10%, which corresponds to an increase of 18.2%. In the GHA30 mix, the final setting time was 250 min, and an extension of 51.5% was observed compared to the reference mix. This increase in the final setting time is related to the decrease in the hydration rate and the structural properties of GHA. The loss on ignition (LOI) rate of GHA (27.6%), i.e., the decomposition of organic and carbonated substances present in it, reduces the reaction rate during the initial hardening stages of the binder matrix. Therefore, the formation of late hydration products is delayed, and the setting takes longer. Similarly, it has been reported that waste ash additives with high organic content cause a delay in setting time, but this may provide a workability advantage in some applications.^42^

The observed prolongation of setting times with increasing proportions of GHA may be an advantage for applications that want to increase the workability time of the mixture. Such delays may be desirable, especially in hot climatic conditions or large-volume castings. However, a delay will disadvantage applications that require early strength gain. Therefore, the substitution ratio should be carefully determined according to the targeted application area when working with agricultural waste admixtures such as GHA. These effects on setting time also support the idea that GHA is not inert but a pozzolanic admixture that reacts with time. The increase in setting times is generally due to the low calcium content of agricultural wastes and late-onset pozzolanic reactions. For example, increased initial and final setting times were observed with POFA substitution.^43^ The coarse-grained, unburnt fibrous structure of agricultural wastes such as POFA can increase the setting time by increasing the water/binder ratio. A similar situation of this effect may be valid for GHA. In studies on RHA and sugar cane bagasse ash (SCBA), setting time increased when the specific surface area of the material was increased by grinding, and the final setting time of cakes prepared with ground RHA increased by 80 min. This was because porous particles with high water absorption capacity delayed hydration by reducing the effective water/binder ratio.^44^ As a result, the high silica and organic matter content and low CaO content of GHA, like similar agro-waste admixtures, led to slow hydration reactions and prolonged setting time.

In Figure 7a, the consistency of the water amounts in the pastes is also presented. For the reference mixture, GHA0, the determined viscosity ratio is 29.8%. At 10% GHA substitution, this increases to 35.4%, representing an increase of approximately 18.8%. When GHA reached 30%, the viscosity increased to 44.8%—a rise of 50.3% compared to the reference mix. This trend is directly related to the high porosity, low density and large specific surface area of GHA. These physical properties cause the binder material to absorb more water, resulting in the mix requiring more water to become workable. GHA is an agricultural waste of organic origin and contains cellulosic residues. Therefore, its particles are porous and have high water absorption capacity. In the literature, it is reported that high surface area agricultural waste additives, such as RHA, increase the consistency of water.^45^ The amorphous and semicrystalline phases of GHA (e.g., carbonaceous residues) can form nonreactive structures that physically retain water. This reduces the effective w/b ratio in the mixture, and more water is needed to compensate for this deficiency. Since GHA, which has a lower CaO content than cement, delays the formation of hydration products, the initial cohesive structure of the matrix is formed later. Therefore, more free water is needed for the formation of consistency. It has been reported that agricultural wastes such as POFA (Palm Oil Fuel Ash) increase viscosity due to their coarse, fibrous, and porous structure, which is proportional to the replacement rate.^46^ As the GHA ratio increases, this increase in the amount of water required for the mixture to thicken affects the rheological balance of the mix. While the rise in thickening water requirement may positively impact the workability, excessive free water use may lead to problems such as high porosity, low strength and tensile deformation. For this reason, it is recommended that water-reducing additives or viscosity regulators be added to GHA-added systems.

The relationship between the setting time and the viscosity showed that with an increase in the GHA substitution ratio, both setting time and viscosity significantly increased. The consistency water to binder ratio, which was 29.8% in the GHA0 reference mixture, became 44.8% in the GHA30 mixture; other parameters include the initial setting time, which increased from 115 to 180 min. This situation reveals that the increase in thickening water prolongs the setting times. Increasing the amount of water of consistency decreases the frequency of contact between cement and GHA particles. It causes a delay in hydration reactions, which prevents the matrix from forming a stable structure at the beginning and prolongs the initial and final setting times. In particular, the porous and high surface area structure of GHA causes the mixture to require more water. At the same time, its low CaO content and late onset of pozzolanic activity delay the formation of hydration products. Similarly, the literature has reported that agricultural waste additives such as POFA, RHA, and SDA cause prolonged setting times. This is associated with high water absorption capacity and low early wet reactivity. Therefore, to control the setting time in GHA-added systems, it is recommended that the thickening water requirement be carefully evaluated and optimized with chemical additives when necessary. Ashes from agricultural wastes such as Phragmites, rice husks, and sugar cane bagasse contain pozzolanic properties that increase water retention and change the microstructure of cement pastes, resulting in prolonged setting times and increased consistency in water absorption.^47^

Figure 7b shows the soundness values of the cement pastes. Le Chatelier expansion testing is a key method for verifying whether cementitious systems exhibit volumetric stability and whether late hydration products will cause expansion that can be detrimental. The expansions shown are directly related to the hydration of those expansion-causing compounds, mainly free lime (CaO) and magnesium oxide (MgO). While the expansion value was 3 mm in the reference mixture GHA0, this value increased to 8 mm in the GHA30 mixture. This reveals that GHA30 shows approximately 166.7% more expansion than GHA0. GHA, due to its high porosity and fine particle structure, causes more absorption of water added to the mixture. Such a phenomenon can create the possibility of an expansion owing to the sluggish reaction of the excess trapped water during the hydration process. The GHA contains oxides such as MgO (2.4%) and CaO (26.3%), which could promote possible expansion. Some of these oxides may be as unburned lime or free in GHA. Such unstable phases are frequently seen, especially in agricultural waste ashes obtained at low combustion temperatures. In addition, the high LOI value of GHA (27.6%) may also have been practical in this process. The high LOI value indicates that the material contains much unburnt organic matter or carbonate content. The gas phases produced after combustion can trigger volumetric expansion during hydration.

Expansion tendencies increase when GHA is used instead of cement. However, the values obtained (3–8 mm) fall below the expansion limit values of 10 mm specified in standards such as TS EN 196-3 and ASTM C151. This means that GHA replacement in the place of cement is volumetrically stable, but its expansion behavior needs to be controlled when used in large amounts, i.e., 30% and above. Pretreatment (calcination, grinding, stabilization) is recommended, especially for agricultural waste admixtures with high LOI values. Alternatively, the volumetric stability of the system will be maintained with low substitution rates.

MgO hydrates to form brucite, which contributes to volume expansion. The degree of hydration directly affects the expansion rate, and higher hydration leads to more expansion.^48^ Furthermore, the anisotropic crystallization of MgO increases the porosity of the matrix by creating internal pressure, leading to further expansion.^49^ Free lime can react with MgO and affect the overall expansion properties. Free lime can enhance hydration, potentially expanding more significantly.^50^ These conditions mentioned in the literature explain the expansions caused by GHA.

#### 3.2.1 Fresh State Properties

Figure 8 shows the fresh state properties of the mixtures with and without GHA content. The data obtained for the flow diameter of the mixtures reveal that the workability decreases with the increase in the GHA substitution rate. While the flow diameter determined for the reference mixture GHA0 was 181 mm, this value decreased to 157 mm in the GHA30 mixture. This decrease corresponds to a regression of approximately 13.3%. The main reason for this situation lies in the physical and rheological properties of GHA. Since GHA has a high specific surface area and porous structure, it physically absorbs a significant portion of the free water in the mixture. This reduces the flowability of the mix and decreases the flow diameter.

**Figure 8:**
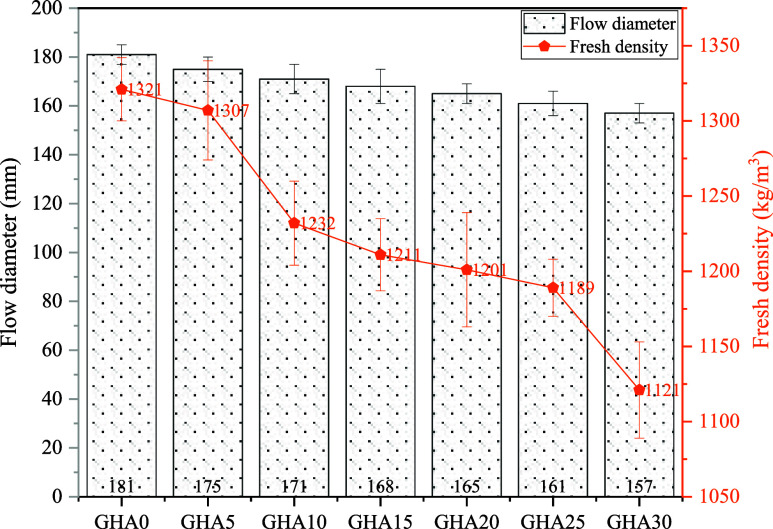
Flow diameters and fresh densities for samples.

Also, the irregular surface texture and angular shape of GHA particles promote interparticle friction among particles in the matrix for a greater rigidity. Based on these observations, one may conclude that the addition of GHA significantly decreases workability, particularly in terms of rheological behavior. Therefore, optimizing the chemical additives or the mixing water ratio is necessary to compensate for the loss of fluidity in systems where the GHA ratio is increased. Another essential criterion affecting the workability is the LOI content of the GHA. Organic residues and porous structures physically trap water in the system. This reduces the amount of ’free water’ in the mixture and the flowability. Therefore, ashes with high LOI require more water, which reduces the flow diameter. The retention of burnt or partially burnt organic components in the mix as insoluble phases can disrupt the homogeneity of the binder matrix. This increases rheological resistance, leading to a thickening of the consistency. Ash additives with high LOI may contain volatile gases, which may impair foam stability during mixing. This is another factor that reduces workability. In addition, it should be considered that the particle structure of GHA in foam concrete systems can harm foam stability. The sharp-edged particles of GHA can weaken the walls of foam bubbles and disrupt the integration of air bubbles in the mix. This suggests that the loss of workability depends not only on water content but also on rheological and microstructural effects. Therefore, the admixture’s water content, particle morphology, LOI level, and physical activity should be carefully evaluated to achieve optimum flowability and workability in GHA-blended systems.

The opposite results have been observed in the literature. For example, the workability of mixtures containing RHA varies according to particle size and grinding time. RHA’s porous structure disintegrates as the grinding time increases, reducing the water demand and making the mixture more fluid. However, since RHA with a high surface area prevents agglomeration by electrostatic interaction with cement particles, less mixing water is trapped, and workability increases.^51^ Using bamboo leaf ash (BLA), corn cob ash (CCA), RHA, and SCBA as partial cement replacements reduced the workability of concrete. The finer particle size of these ashes explains this. Since fine particles have a higher surface area, they increase the water requirement and reduce the workability.^52^ In other studies in the literature, it has been reported that agricultural wastes and ashes obtained from these wastes generally deteriorate workability.^53–55^ Hence, the influence of agrarian waste ashes on cementitious composites’ workability is typically detrimental. This influence can be enhanced through additives or a suitable mix design. Despite the damaging workability effect, the environmental advantages of their utilization and the pozzolanic activity of these ashes render their use in cementitious composites appealing.

Although visual documentation was not provided for all mixes, GHA10 and GHA30 mixes were selected explicitly for comparative visual presentation because they represent the extreme limits of workability performance. As seen in Figure 9, GHA10 exhibited a more extensive flow diameter due to the lower replacement rate, while GHA30 exhibited a more viscous and cohesive structure with lower flow. These photographs are provided to support the quantitative workability data visually.

**Figure 9:**
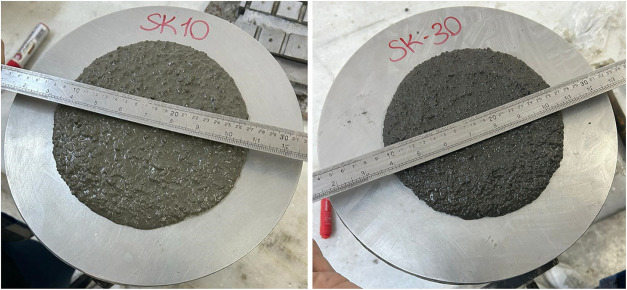
Flow diameters of GHA10 and GHA30 mixtures.

According to the data obtained, while the fresh unit volume weight of the GHA0 reference mix was 1321 kg/m^3^, this value decreased to 1121 kg/m^3^ in the GHA30 mix (Figure 8). This decrease shows that there is a density decrease of approximately 15.1% between GHA30 and GHA0. This decrease is due to the physical properties of GHA and its effects on the mixture. GHA is a lighter material compared to cement. Due to its chemical composition and structural porosity, a lighter binder phase is added to the mix by mass when GHA is substituted for cement. This reduces the total mass of the mixture and decreases the unit volume weight. GHA can absorb a lot of water, which means that more water was needed for the mix (from 29.8% to 44.8% in the previous test results). This increase may have resulted in more air voids in the fresh concrete mix or a more prolonged stabilization of the existing foam. The more remarkable preservation of the foam structure causes the fresh concrete to be less compact and dense. This accelerates the decrease in fresh density. The observed reduction in the flow diameter data indicates that the workability of the mixture decreases, resulting in a more irregular particle distribution and more entrapped air during molding. This leads to a more porous structure of the matrix and a decrease in fresh density. As a result, GHA replacement systematically reduced the fresh density of foam concrete. This reduction is directly related to the physical structure (low density, porosity), chemical content (low CaO, high LOI), rheological effects (high need for thickening water), and air retention tendency of GHA. This result shows that GHA admixed foam concretes are suitable for producing lightweight construction materials. However, this density reduction must be evaluated (optimized) with other engineering properties, such as strength. Agricultural waste ashes generally have a lower specific gravity and finer grain structure than cement.^52^ Therefore, they tend to reduce the overall density of the mix when used as a partial replacement for cement.

Furthermore, these ashes’ high water absorption capacity and large surface areas tend to retain more water in the mix.^52,56^ This increases the volume of the fresh composite and reduces its density. The reducing effect of agricultural waste ashes on the fresh density of cement-based composites is mainly due to their low specific gravity, fine-grained structure and high water retention capacity. These properties increase the volume of the mixture while decreasing its weight, resulting in a decrease in fresh density.

#### 3.2.2 Physical Properties

Figure 10 shows the physical properties of the samples with and without GHA content, which gives information about porosity. When the apparent porosity values are analyzed (Figure 10a), the porosity ratio, 24.11% in the GHA0 mixture, reaches the highest level of 32.12% in the GHA10 mixture and then decreases to 24.96% in the GHA30 mixture. This change shows that the effect of GHA admixture on the microstructure of the mix is not linear; the porosity increases up to a specific replacement rate and then decreases again. First, the significant increase in porosity when GHA was substituted up to 10% (24.11% → 32.12%) can be attributed to several main reasons. The high specific surface area and porous nature of GHA resulted in more water being trapped in the mixture, which became less compact as a result. In addition, the low reactivity of GHA prevented the formation of sufficient binder gel in the matrix at an early age, contributing to forming a porous structure. A gradual decrease in porosity values was observed when the GHA substitution rate increased above 10%. In the GHA30 mixture, a porosity value of 24.96% was obtained, which is very close to the initial porosity. This can be attributed to the activation of the pozzolanic activity of GHA at later ages. Increasing GHA content, especially in the range of 15–30%, may have led to the formation of more secondary gel products (e.g., C–S–H) in the internal structure of the matrix. These secondary reactions partially filled the porous areas, thus reducing the porosity. The previous flow diameter results also correlate with this finding: The flow diameter decreased for mixtures with GHA10 and above (GHA10:171 mm → GHA30:157 mm), i.e., the mixture became more viscous. This may have helped to develop a more compact structure by reducing the tendency of the mixture to form voids during casting. The high LOI value of GHA (27.6%) indicates that the ash added to the mixture carries organic and carbonaceous content. These contents may increase the porosity by generating outgassing during the hydration process. However, it is thought that this effect stabilizes as the GHA ratio increases and the impact of some unburnt components decreases. Therefore, while the porosity increase was evident up to the first 10% substitution, the ash acted more like a pozzolanic additive at higher ratios and decreased the porosity. Porosity in foam concrete systems depends not only on binder properties but also on the stability of air bubbles. The sharp-edged grains and low fluidity (reduction in flow diameter) of GHA can make it difficult for the air bubbles to disperse more homogeneously, leading to uneven pore distribution in the initial stage. However, at higher GHA ratios, this structure stabilized, and the air distribution in the mix became more controlled.

**Figure 10:**
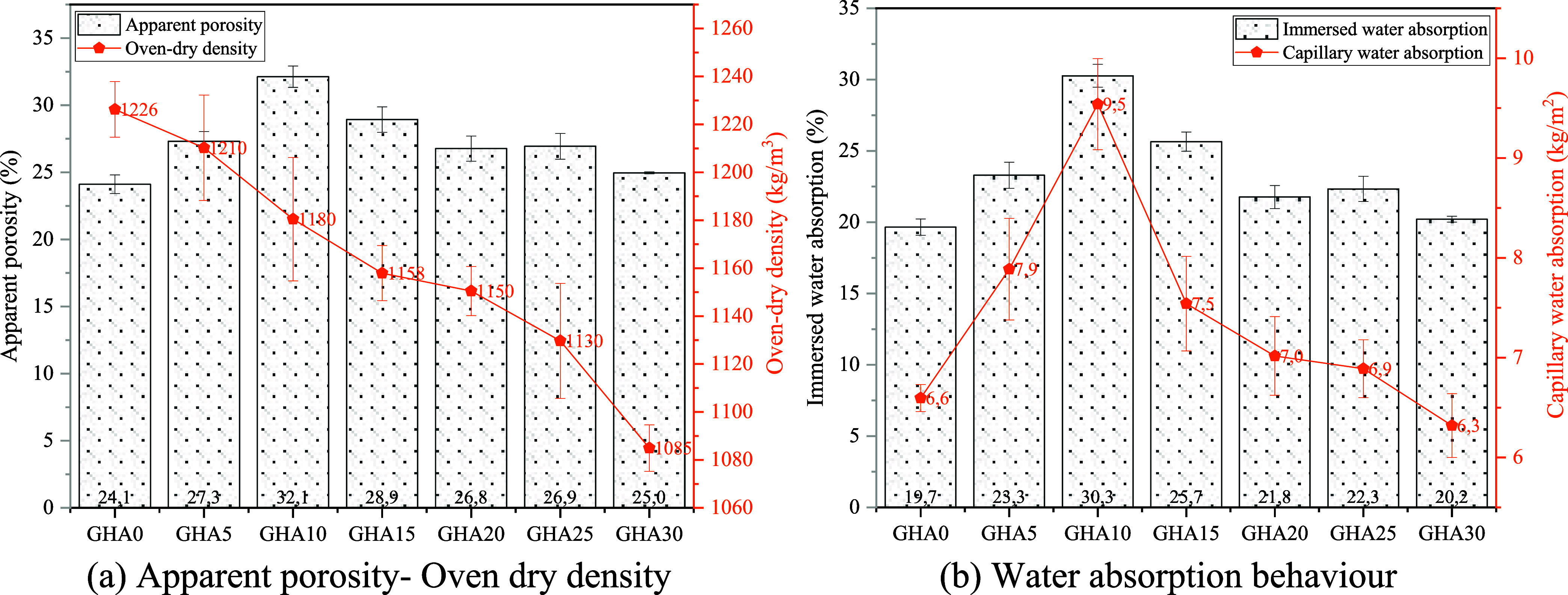
Physical properties of samples.

Apparent porosity values show that GHA addition can have positive and negative effects. At low substitution ratios, porosity increased due to workability deterioration and lack of early wet binder, whereas at high substitution ratios (GHA20-30), porosity decreased due to improved pozzolanic reactions and compacting. This indicates that the optimum utilization range of GHA should be carefully determined in terms of performance. In addition, it is essential to evaluate all parameters, such as density, consistency, and setting time, together in GHA admixed foam concretes to accurately control microstructural properties, including porosity. Adding agricultural waste ashes improves durability and strength by reducing the total pore volume in cement composites. For example, RHA has been shown to reduce permeability and capillary water absorption, leading to lower porosity levels.^57^ Recently completed studies have reported that replacing 5–15% of the cement with RHA or 5–30% with SCBA reduces porosity.^58,59^ The porosity reduction mechanism of these ashes is explained as follows: the pozzolanic nature of agricultural waste ashes contributes to the formation of additional calcium silicate hydrate (C–S–H), which fills the voids and reduces porosity.^57^ Scanning electron microscopy shows that incorporating agricultural waste ashes into the matrix produces a denser microstructure, showing better adhesion and fewer voids.^57^ While the benefits of using agricultural waste ashes in cementitious composites are clear, some studies show that excessive replacement can lead to increased permeability and reduced mechanical performance if not carefully managed.^60^ This indicates the need for balanced compounding strategies to optimize the benefits of GHA in construction materials.

The oven dry density values show a significant decrease as the GHA content increases (Figure 10a). The dry density value of 1226 kg/m^3^ in the GHA0 mixture decreased to 1085 kg/m^3^ in the GHA30 mixture. This indicates a decrease of approximately 11.5%. This suggests that GHA admixture directly affects the mass-volume relationship of foam concrete and strengthens its lightweight construction characteristic. The primary reason for this reduction is that GHA has a lower specific gravity than cement. Regarding chemical composition, GHA contains a relatively high amount of amorphous silica (SiO_2_) and is characterized by a low CaO content. This lightens the total mass of the binder system. At the same time, the particle structure of GHA is more porous and has a lower density than cement. This means that each unit of GHA substituted adds less mass to the mix. The oven dry density data coincide with the fresh unit volume weight and apparent porosity data obtained previously. For example, the fresh density for GHA0 is 1321 kg/m^3^, and the oven-dried density is 1226 kg/m^3^, while for GHA30, these values are 1121 and 1085 kg/m^3^, respectively. This similar decrease curve reveals that GHA admixture decreases the density and increases the porosity in both fresh and hardened structures. Porosity data showed an increasing trend up to GHA10, and a decreasing trend was observed. This indicates that when the binding ability of GHA is poor at an early age, more voids are formed in the system; however, at later substitution rates, the reactive silica additive starts to make the microstructure more compact. This intrastructural stabilization effect also stabilizes the oven-dry density within a specific range.

The oven-dry density values show that the GHA admixture allows foam concrete to become a lighter construction material. This is a significant advantage for applications such as thermal insulation, lightweight structural elements, and floor and roof systems where load reduction is crucial. These risks may include losing strength, increased water absorption, and weight loss. GHA admixture utilization can be evaluated in maximum detail regarding density, durability, and mechanical performance. In addition, the application of agricultural waste ashes, such as bagasse, in concrete production will probably result in decreased densities since these ashes are generally much lighter than the conventional aggregate and cement and, thus, contribute more to the lightweight properties of concrete.^61^ Incorporation of agricultural waste ashes, such as RHA and POFA, into foam concrete reduced bulk density. The study indicated that the bulk density decreases with increasing RHA and POFA substitution levels (up to 20 and 30%, respectively). The reduction in density is due to the lower specific gravity of agricultural waste compared to concrete, or is attributable to the lightweight nature of the concrete overall.^29^

The immersed water absorption values vary according to the GHA admixture ratio, especially at a 10% substitution rate (GHA10), reaching the highest value (30.27%) and then decreasing gradually (Figure 10b). This indicates that the effects of GHA admixture on the void structure and water absorption tendency of micropores in foam concrete occur through a dual mechanism of action. The immersed water absorption rate, which was 19.65% in the GHA0 mixture, increased to 30.27% in the GHA10 mixture. This corresponds to an increase of approximately 54%. The main reason for this increase is that the low GHA additions cause the formation of more capillary voids in the internal structure of the mixture. This agrees with the previous porosity data: The apparent porosity in GHA10 was the highest at 32.12%. At the same time, the high water consistency requirement (35.4%) and low flow diameter (171 mm) of the GHA resulted in the mix containing more free water and being less compact. This led to the formation of a more permeable microstructure that absorbs water quickly.

As the GHA substitution rate was increased above 10%, a gradual decrease in the water absorption rates was observed. The water absorption value of the GHA30 mixture decreased to 20.21%, almost close to the reference mixture (GHA0). This decrease can be attributed to an increase in the GHA ratio, which triggers pozzolanic reactions over time. The high silica content of GHA contributed to the formation of more C–S–H gels at increasing substitution ratios, resulting in a more compact microstructure by closing/reducing some of the capillary voids. This interpretation is also supported by the previous porosity and oven-dry density data: In GHA30, the porosity decreased to 24.96%, and the oven-dry density decreased to 1085 kg/m^3^. The denser and less voided structure limits the diffusion of water into the specimen and reduces the water absorption. The particle structure and porosity of GHA initially lead to the absorption of more free water, whereas at higher replacement rates, this effect is replaced by an improved binder structure. In addition, the high LOI (Loss on Ignition) value may cause the formation of gas voids in the sample at initial substitution rates. However, the effect of these voids decreases over time; pozzolanic reactions stabilize the structure, and a less absorbent structure is formed.

The water absorption behavior of GHA admixed foam concretes first increases and then tends to decrease depending on the substitution rate. This shows that the rheological effects of GHA increase the porosity in the first stage. Still, its pozzolanic character improves the microstructure at higher proportions and reduces the water absorption rate. For structures at risk of water exposure, the optimum contribution rate of GHA should be 15–30%, which can provide both lightweight and low water permeability. These effects of GHA offer promising results in the goal of developing sustainable and durable building materials.

According to the capillary water absorption results, the capillary water absorption increased significantly as the GHA additive ratio increased up to 10% and then showed a decreasing trend from 15% onward (Figure 10b). The capillary water absorption value, which was 6.60 kg/m^2^ in the GHA0 reference mix, reached a peak of 9.54 kg/m^2^ in GHA10 and decreased to 6.32 kg/m^2^ in GHA30. These results reveal that GHA affects the capillary absorption behavior of foam concrete through a dual mechanism. Capillary water absorption is directly related to the concrete’s volume and continuity of capillary voids. When GHA admixture is substituted at low levels (5–10%), a significant increase in the viscosity water content (29.8% → 35.4%) and the decrease in the flow diameter (181 mm → 171 mm) indicate that the mix becomes less fluid, more irregular and prone to air void formation. This leads to the formation of more open and interconnected capillary voids. In addition, since the pozzolanic effect of GHA is not yet activated at these substitution levels, the impact of pore closure or filling in the microstructure remains limited. As a result, capillary water absorption increases significantly. The 44% increase observed in the GHA10 mixture is significant in this context. When the GHA substitution rate exceeded 15%, capillary water absorption rates tended to decrease. In the GHA30 mixture, this rate decreased to 6.32 kg/m^2^, reaching a value even lower than GHA0. This indicates that secondary pozzolanic reactions became effective with a high GHA content. The silica component of GHA (25.8%) reacts with calcium hydroxide to yield additional C–S–H gel, which partially fills the capillary voids, lowering permeability and water absorption. This observation agrees with the downward trend of apparent porosity (32.12% → 24.96%) and water absorption (30.27% → 20.21%) values assessed previously. Underlying this is that capillary water absorption operates not just on the count of voids, but also, just as critically, on the continuous-void network systems with a specific size distribution. The capillary water absorption test states that water movement along the material’s surface depends on the number of pores and whether those pores are interlinked (permeability). A high GHA admixture can narrow water gateways by reducing microporous voids. In mixtures such as GHA30, this limits the advancement of water, resulting in low capillary absorption values.

The capillary water absorption behavior of GHA admixed foam concretes varies according to the substitution rate. Admixtures up to 10% increase water absorption by forming a porous and permeable structure; however, at higher admixture levels, the structure becomes denser, and permeability decreases due to pozzolanic reactions. This demonstrates that GHA is an environmentally friendly binder alternative and an effective mineral admixture that enhances waterproofing performance. It is recommended to use GHA admixture in the range of 15–30%, especially in lightweight structural elements that will work outdoors and have the risk of exposure to water. It is seen that the relationship between capillary water absorption and immersed water absorption is parallel to each other. In general, agricultural waste ashes improve the water absorption properties of cement-based materials. For example, it has been observed that water absorption decreases when SCBA is substituted for cement at a rate of 15%. This improvement is due to the densification of the concrete’s microstructure due to the ash’s pozzolanic reactivity.^62^ The effect of agricultural waste ashes on water absorption behavior depends on the type, quantity, and physical properties of the ash used. For example, materials such as BLA, CCA, RHA and SCBA can affect the water absorption properties of concrete due to their finer particle size and high surface area.^52^ The pozzolanic activity of agricultural waste ashes causes secondary reactions in the cement matrix, resulting in a more dense and uniform morphology. This leads to the condensation of the hydrated gel and, thus, an increase in the water absorption resistance of the concrete.^62^

In conclusion, the effect of agricultural waste ashes such as GHA on the water absorption behavior of cementitious composites is generally favorable. However, selecting the appropriate type and amount of ash for optimum performance and considering the material’s physical and chemical properties is essential. This approach will contribute to developing environmentally friendly and sustainable building materials.

#### 3.2.3 Mechanical Properties

##### 3.2.3.1 Compressive Strength

When the compressive strength results of GHA admixed foam concretes were analyzed, a very characteristic change was observed depending on the replacement rate and curing time (Figure 11a). While GHA0, the reference mix, showed the highest compressive strength at early ages (10.76% - day 3), this value showed a steady but limited increase over time and reached 12.65 MPa at the end of 90 days. In the mixtures containing GHA admixture, it was observed that the early age strengths decreased significantly, especially in the groups with up to 10% substitution rate. For example, the GHA10 mix could only reach a value of 4.54 MPa on day 3, representing a decrease of approximately 58% compared to the reference mix. This significant decrease is explained by the low calcium content, high amorphous silica content, and incredibly high LOI value of GHA. The high LOI value adversely affects the early age strength as unburnt organic residues in the matrix inhibit hydration.

**Figure 11:**
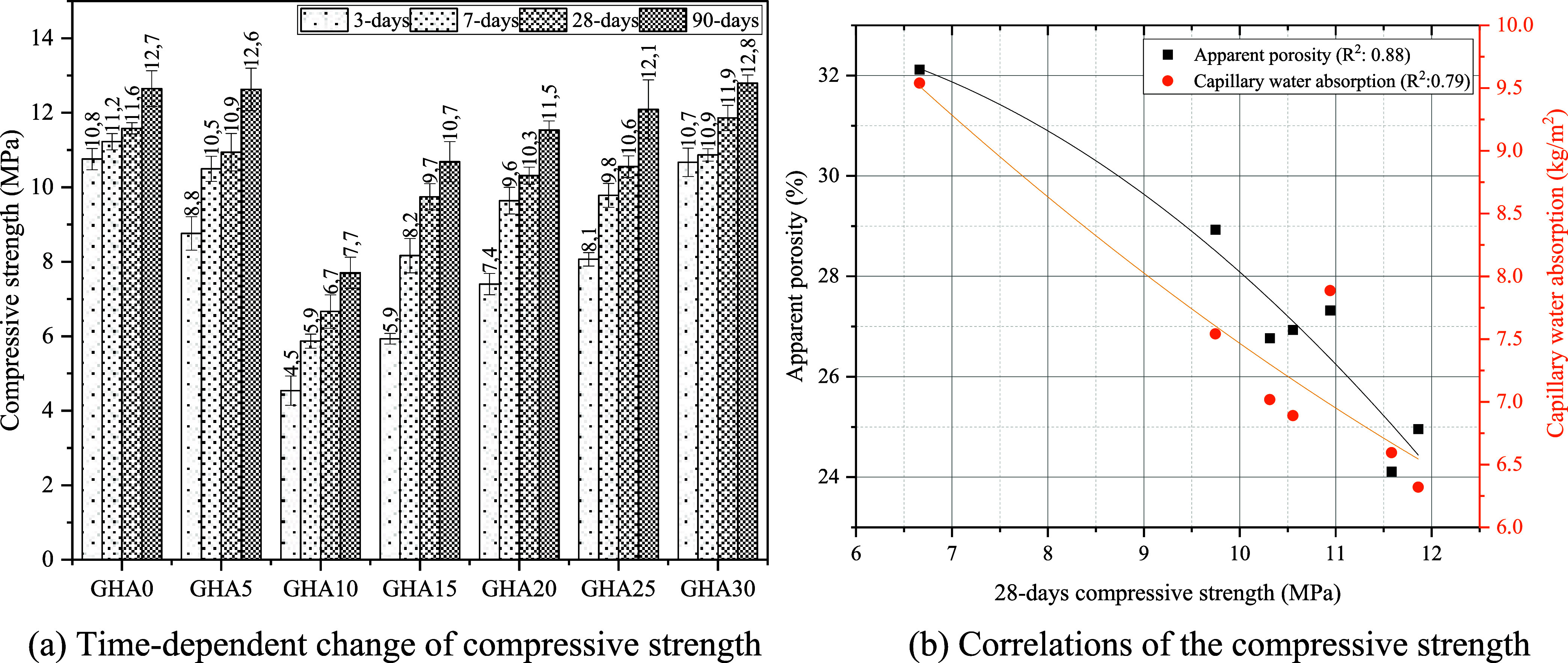
Compressive strengths of specimens.

Interestingly, a recovery in strength was observed over time, especially at GHA replacement ratios in the range of 20–30%, and in some cases, even higher values than the reference mix were obtained. The GHA30 mix reached the highest long-term compressive strength of 11.86 MPa at 28 days and 12.80 MPa at 90 days. This development is a result of the late-age pozzolanic activity of GHA. The reactive amorphous silica in GHA reacts with the calcium hydroxide released as a result of the hydration of the cement over time, increasing C–S–H gel formation and making the internal structure of the matrix more compact. This mechanism supports the strength increase observed in the GHA30 mixture. This is also consistent with the fact that the water absorption (20.21%) and porosity (24.96%) values of GHA30 are very close to the reference mix levels. The increase in matrix density and the decrease in microcracks and voids directly increase the load-carrying capacity.

When the differences between early age and late age strengths are analyzed, it is seen that GHA admixture significantly affects the timing of in-matrix reactions and microstructure development. The strength gain rate from the third to the 90th day is relatively high in GHA blended mixtures. For example, the GHA10 mixture showed a 69% strength gain from the third to the 90th day. This rate is around 17% in the reference mix. This indicates that although the binding property of GHA admixture is limited in the first place, it increases its binding property over time. In other words, the contribution of GHA to mechanical performance was delayed but effective.

The results obtained in this study reveal the indirect but intense effects of machinability parameters on compressive strength. When the flow diameter values of the GHA blended mixtures were analyzed, while the GHA0 mixture was 181 mm, this value decreased to 157 mm for GHA30. This decrease shows the mixture has gained a more viscous and difficult-to-settle structure. This decrease in the flow diameter is related to the particle structure of the GHA additive, which has sharp edges, roughness, and a coarse surface. This physical structure changed the rheological properties of the mixture and reduced the workability. This deterioration in workability prevents the fresh mix from settling correctly in the mold, resulting in air voids, lumps, or noncompact zones. This situation adversely affects mechanical performance, especially at an early age.

Indeed, the third-day compressive strength results indicate serious mechanical weaknesses in mixtures with low workability. For example, the GHA10 mix is one of the groups with the lowest flow diameter values and 3-day strength (4.54 MPa). This can be explained by the inability of the mixture to settle sufficiently in the mold due to the thickening of the consistency. In addition, this viscous structure creates a disadvantage in terms of mold vibration or air escape and causes the development of an inhomogeneous microstructure in the matrix. On the other hand, it is observed that this negative effect is partially stabilized or eliminated at later ages. The main reason for this is the pozzolanic effects of GHA at late ages. In other words, although poor workability limits the mechanical strength in the first days, secondary gel formations (especially C–S–H), which occur with pozzolanic reactions on the 28th day and later, partially fill the gaps and increase the late age strength by densifying the internal structure of the matrix. Therefore, the workability-concrete strength relationship is more substantial and decisive at early ages and balanced by secondary reactions at late ages.

As a result, although GHA admixture slightly decreases the early age strength of foam concretes, it provides significant recovery in the long term and even reaches higher strength values than the reference mix in some proportions. This demonstrates the strong late-age pozzolanic effect of GHA and its significant contribution to the development of second-order binder phases in the foam concrete matrix. In particular, 25–30% replacement ratios can maintain competitive levels in compressive strength at an early age and support strength increases at a late age. This demonstrates that GHA, as a sustainable mineral admixture, is a technically feasible and environmentally valuable alternative in foam concrete production.

Figure 11b shows that apparent porosity and capillary water absorption values decrease systematically as the 28-day compressive strength increases. The correlation coefficients indicated on the graph are also exceptionally high: R^2^ = 0.83 for apparent porosity and R^2^ = 0.79 for capillary water absorption. This shows that as the porosity structure of foam concrete develops, the compressive strength decreases, and the permeability increases with it. In mixtures with high porosity, the matrix is less dense, the microvoid ratio is higher, and there are discontinuities between the binder phases. This limits the load-carrying capacity. Likewise, the more straightforward progression of water through this void structure increases capillary water absorption values. Especially low-strength mixtures, such as GHA10, have high porosity (approximately 32%) and high capillary water absorption (9.54 kg/m^2^). These mixtures also showed low early age strength (day 3:4.54 MPa), confirming that the microstructure was not sufficiently packed with binder gel and remained hollow. On the other hand, mixtures with a denser matrix, such as GHA30, which showed high strength at late age (90th day: 12.80 MPa), decreased below 25% in apparent porosity and capillary water absorption up to 6.32 kg/m^2^. This indicates that GHA enhances the microstructure through its pozzolanic effect, which is activated at a late age, providing advantages in terms of strength and impermeability. The nonlinear relationship observed between capillary suction and strength suggests that this parameter is related to the number of pores and the continuity and size distribution of the pores. Furthermore, this graph strongly suggests that foam concretes’ strength and durability performances are directly related to their microstructural properties. Parameters such as apparent porosity and capillary water absorption are permeability indicators and essential determinants of mechanical performance. By using GHA admixture in appropriate proportions, both porosity and permeability can be reduced, and strength can be increased. The graph shows that this optimum balance is achieved, especially at 25–30% substitution rates.

Studies have shown that ashes obtained from agricultural wastes can increase compressive strength. In a survey of the evaluation of Oil Bean Husk Ash (OBHA) and Bread Fruit Husk Ash (BHA) as cement replacement materials, it was stated that the addition of these ashes at a rate of 15–20% by weight would increase the compressive strength. OBHA would generally perform better than BHA.^63^ Used as a partial replacement for Ordinary Portland Cement (OPC), SCBA has shown excellent compressive strength improvements in concrete, especially when combined with Ground Granulated Blast Furnace Slag (GGBFS).^64^ Optimum substitution levels of 5% RSA (Rice straw ash) in concrete mixes showed superior mechanical properties, especially when combined with recycled coarse aggregates.^65^ It has been reported that ash from agricultural waste reacts with calcium hydroxide produced during cement hydration to form additional calcium silicate hydrates that increase the strength and durability of the concrete matrix.^66^ Incorporating these ashes has been reported to lead to denser microstructures, reducing porosity and increasing resistance to harmful environmental factors.^64^ While the benefits of using agricultural waste ashes are clear, some studies suggest that excessive substitution can reduce strength due to insufficient cement content and emphasize the need for optimum proportions in concrete mixes.^66^ It shows that replacing up to 50% of the cement with CCA in foam concrete containing CCA can provide compressive strengths above 29 MPa, significantly higher than conventional mixes.^34^ Ashes from agricultural wastes, especially RHA and POFA, improved the compressive strength of lightweight foamed concrete by improving the microstructure and bonding properties, and optimum results were obtained at 20% RHA and 30% POFA substitution levels.^29^

##### 3.2.3.2 Flexural Strength

The reference mix without GHA admixture (GHA0) showed above-average flexural strengths at all age intervals, reaching values of 1.09 MPa at day 3, 1.42 MPa at day 28, and 1.68 MPa at day 90 (Figure 12). This result shows that CEM II A-LL 42.5N cement provides high binding strength at early ages, allowing the matrix to gain a crack-resistant structure. The flexural strength decreases observed early in most GHA admixtures are remarkable, especially in groups up to a 10% replacement ratio. For example, the third-day flexural strength of the GHA10 mixture was only 0.77 MPa, representing a decrease of approximately 29.4% compared to the reference. This decrease can be explained by the low early reactivity of GHA, its relatively low CaO content (compared to pozzolans such as blast furnace slag), and its high LOI, which inadequately supports the formation of a binder gel that provides crack resistance to the matrix.

**Figure 12:**
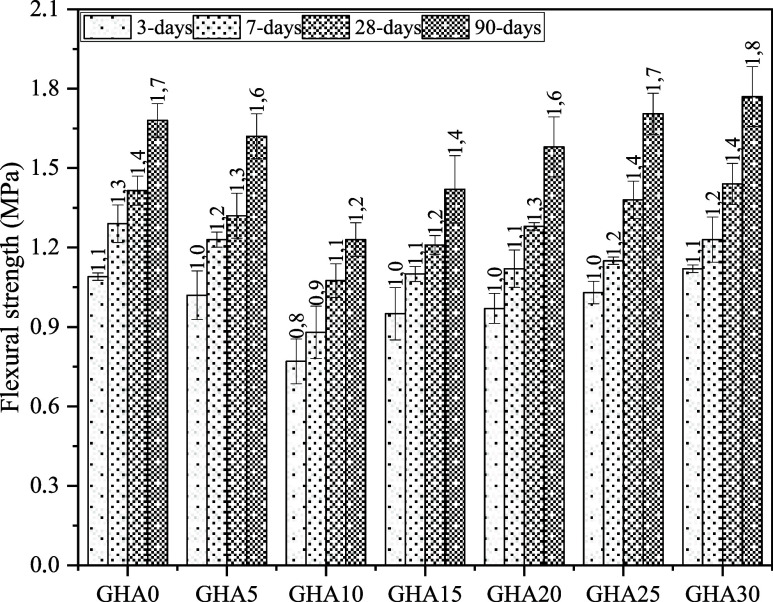
Flexural strength results.

With time, the positive effects of GHA admixture became more pronounced, especially in the 28th and 90th day strengths. GHA25 and GHA30 mixes outperformed the reference mix by showing flexural strengths of 1.71 and 1.77 MPa at 90 days. This indicates that the late-age pozzolanic effects of GHA contribute to the formation of more C–S–H gels in the microstructure of the matrix, and these gel networks form a mechanism to inhibit crack propagation. The GHA30 mix also gave the best results in compressive strength, indicating a strong correlation between these two mechanical parameters.

Increased flexural strength has been successfully documented in GHA blended systems, especially over the 7- to 28-day period. The GHA15 mixture gained a flexural strength of 1.10 MPa at day 7 to 1.21 MPa at day 28, while the GHA25 mixture gained from a flexural strength of 1.15 to 1.38 MPa, an increase of about 20%. This means the pozzolanic reactions are slow but effective, with new gel products forming around the microcracks to hinder crack propagation. Such microstructural improvements can make more of a difference, especially in mechanical properties directly related to brittleness, such as flexural strength.

In this context, it can be said that GHA admixture provides positive contributions in terms of crack control and ductility in foam concretes in the long term. Flexural strength is a critical indicator not only of the bearing capacity of the material but also of durability, impact resistance and service life. The positive effect of GHA admixture on 28- and 90-day performances shows that this admixture is an environmentally friendly and sustainable binder alternative and can contribute to the mechanical integrity of foam concretes.

It has been determined that agricultural waste ashes generally have favorable effects on the flexural strength of lightweight concrete or cementitious composites. Mechanical properties are preserved when agricultural waste ashes, such as CCA and SCBA, are used as fly ash replacement in geopolymer concrete.^67^ Similarly, using SCBA to replace up to 15% of cement increased the strength.^62^ In Sun et al.’s study, flexural strength increased between 11.5 and 40.7% in RHA composites.^68^ The mechanism of this favorable effect is attributed to the high silica content and pozzolanic reactivity of agricultural waste ashes. The ashes react with the cement particles to form a dense structure, increasing strength.^62^ In addition, when used in appropriate proportions, these ashes fill the voids in the concrete and provide a more compact structure.

In conclusion, using agricultural waste ashes in optimum proportions generally positively affects the flexural strength of lightweight concrete and cementitious composites. Ash’s pozzolanic properties and microfilling effect can explain this effect. However, it is essential to determine the utilization rate carefully because decreases in strength can also be observed in the case of excessive utilization.^69^

#### 3.2.4 Drying Shrinkage

Drying shrinkage is an essential deformation due to the volume loss of cement-based binder systems, caused by the evaporation of free water in the concrete. While the drying shrinkage value measured at the end of the 120th day in the GHA0 reference mix was 2332 με, the GHA10 mix showed the highest deformation level by reaching 2984 με at the end of the same day (Figure 13a). This reveals that GHA can have an increasing effect on drying shrinkage, especially at some ratios, causing higher volumetric deformations due to water loss on the microstructure.

**Figure 13:**
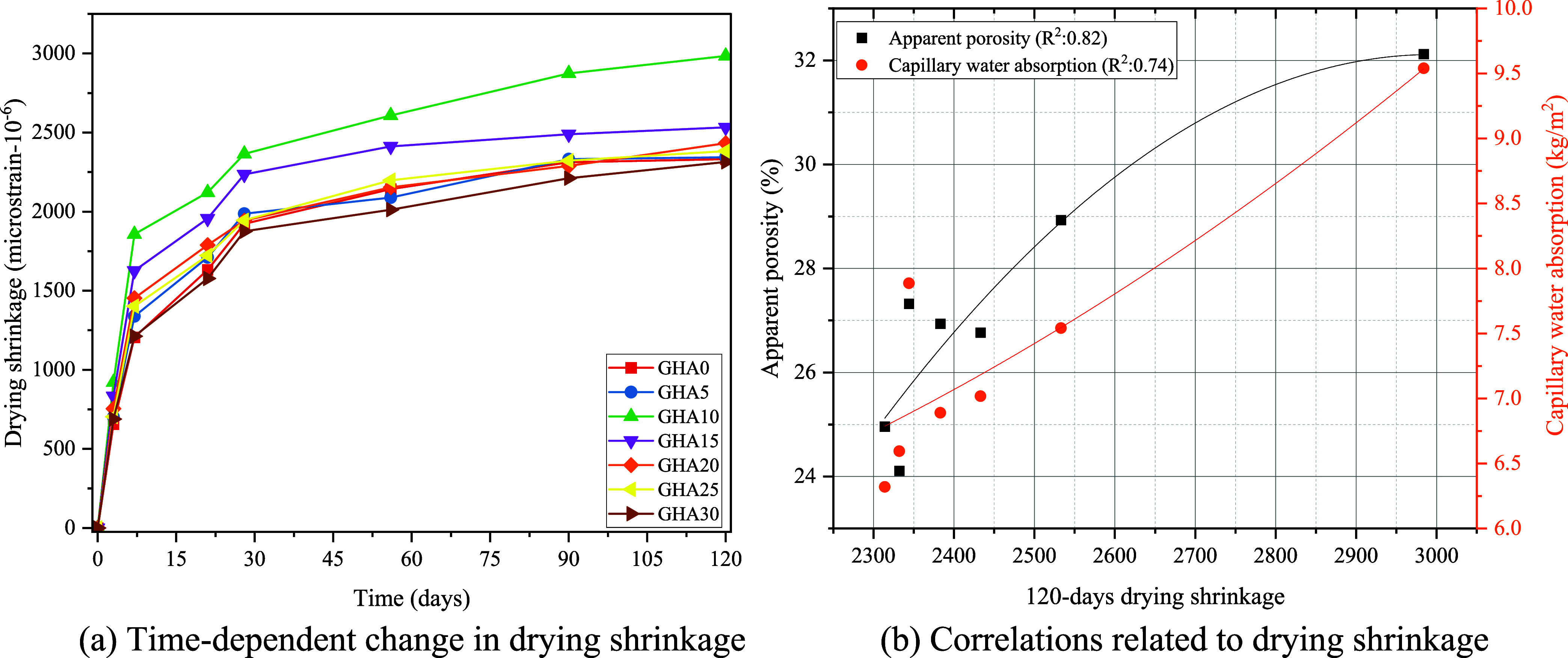
Drying shrinkage of the specimens.

The main reason for this increase lies in the physical and structural properties of GHA. GHA is an agricultural waste with high porosity, fine grain and reactive surface. At the same time, the highest water absorption (30.27%) and porosity (32.12%) values of the GHA10 mixture indicate that this mixture has a porous and permeable structure. The water in this structure evaporates faster and more, which leads to higher drying shrinkage values. On the other hand, GHA25 and GHA30 blends exhibited shrinkage values very close to the reference blend value, with 2383 με and 2314 με at the end of 120 days, respectively. This indicates that as the substitution rate of GHA increases, more binder gels are formed in the microstructure, and these gels provide resistance to deformation by partially filling the capillary gaps. The recovery in the compressive and flexural strength of the GHA30 mixture, especially in the 28–90-day interval, supports the effect of this densified microstructure. Furthermore, the late-onset pozzolanic reactions of GHA resulted in the stiffening of the microstructure at later ages, which in turn limited shrinkage. When the time curves in the graph are analyzed, it is seen that the fastest deformation occurs in the first 28 days in all mixtures. For example, GHA0 reached a value of 656 με at day 3 and 1923 με at day 28, indicating that approximately 82% of the total deformation occurred within the first 28 days. This observation emphasizes that shrinkage in foam concrete systems occurs mainly at early ages, and moisture control is more critical during this period. This behavior is also valid for GHA admixture; however, this rate is spread over a more extended period at high GHA content, and the total deformation is more limited. The binding character of GHA is quite different from that of OPC. Especially at high GHA content, the amount of cement in the mixture decreases, and most of the binding becomes dependent on the late-onset pozzolanic reactions of GHA. While producing a low proportion of C–S–H early, these reactions react with Ca­(OH)_2_ over time and favor additional gel formation at later ages. These delayed reactions gradually densify the microstructure of the matrix, filling some of the pores in the last days and causing the capillary spaces to shrink. Thus, the outward movement and evaporation of water occur more slowly. This contributes to more balanced and limited drying shrinkage over time. The relatively high silica content of GHA (approximately 25.8%) enables new binding gels, especially C–S–H, to form at an advanced age. This gel phase clogs or narrows the capillary waterways by filling the previous voids. This obstruction slows down vapor diffusion and prolongs the time for water to reach the outer surface. Thus, shrinkage reactions spread over time, and sudden deformations are prevented. In other words, when water loss is slower, shrinkage stresses accumulate less suddenly, reducing the risk of crack formation. The fact that GHA contains organic residues (high LOI) may slow the nucleation process of hydration products. Although this may initially appear to be a negative effect, it may lead to the formation of microstructures with lower internal stresses and more homogeneous development. This mechanism contributes to the shrinkage spreading over time instead of sudden jumps.

As a result, the effect of GHA admixture on drying shrinkage varies depending on the admixture ratio and the microstructure properties formed by the admixture in the matrix. At replacement ratios up to 10%, drying shrinkage increases significantly due to increased porosity, water absorption and free water content, while at 25–30% replacement ratios, the pozzolanic reactions of GHA provide filling of voids, densification of the microstructure, and limitation of deformation. This shows that GHA should be used in balanced proportions for strength and deformation control.

Agricultural waste ashes, such as olive waste ash (OWA) and POFA, have been found to modify the hydration process of cement. For example, POFA has been shown to increase long-term compressive strength and reduce drying shrinkage by about 27% due to its pozzolanic activity, which improves the pore structure and reduces water loss.^70^ Similarly, OWA reduces chemical and autogenous shrinkage, but its effect on drying shrinkage varies with the replacement level.^71^ Cement replacement materials, such as ash, have a reducing effect on drying shrinkage because they change the porosity in the matrix. Ash particles filling the voids and forming a denser matrix are a factor that reduces drying shrinkage.^72^ While using agricultural waste ash in cement composites is beneficial in lowering drying shrinkage, it can also decrease mechanical properties, such as compressive and flexural strength, depending on the type and amount of ash used.^71^ Similarly, it is seen that GHA used in this study has a pozzolanic potential due to its high reactive silica content and contributes to the densification of the microstructure, especially at late ages. This densification reduces capillary voids by partially filling the pores in the matrix, thus limiting the drying shrinkage caused by water loss. The limited shrinkage amounts observed in GHA25 and GHA30 mixtures especially reveal that GHA, like POFA, reduces drying deformations through porosity control. In addition, the shrinkage of the voids due to the filling effect of GHA particles and the matrix becoming more rigid may have provided a more balanced deformation distribution. However, as stated in the literature, it can be said that these positive effects of GHA depend on the additive ratio, and the risk of a decrease in mechanical strength at high substitution ratios should be considered. In this respect, GHA can be regarded not only as a sustainable admixture but also as a functional mineral admixture in controlling drying shrinkage.

Figure 13b, both curves are positively sloped, and the *R* ^2^ correlation coefficients are high: *R* ^2^ = 0.82 for apparent porosity and *R* ^2^ = 0.74 for capillary water absorption. In particular, this strong relationship between porosity and drying shrinkage reveals how effective the void volume in the microstructure is on drying-induced deformation. Mixtures with high porosity contain more free water, and as this water evaporates over time, volumetric losses occur. This causes an increase in shrinkage. This is observed in the graph; for example, the GHA10 mix with the highest 120-day shrinkage value (approximately 2984 με) also has the highest porosity value (32.12%) and the highest capillary water absorption rate (9.54 kg/m^2^). This confirms the effect of the hollow and permeable structure to increase the drying deformation. The relationship between capillary water absorption and desiccation shrinkage is also remarkable. Through capillary spaces, water can be transported from inside to outside and absorbed back into the inside from the external environment. However, in the drying process, these voids cause more water to be expelled; the voids left behind by the evaporation of water cause shrinkage of the matrix. Systems with high capillary absorption values become especially vulnerable to losing more water and experience higher deformation with increased internal stresses. This situation can be used to predict the deformation behavior of the microstructure by considering it together with porosity in GHA blends. Ultimately, this correlation graph shows that the microstructure properties are the most fundamental determinant of drying shrinkage in foam concretes. Both apparent porosity and capillary water absorption are strong predictors of shrinkage behavior. Therefore, the deformation control of foam concretes should be considered in terms of binder type or hydration processes in conjunction with void structure and water-structure interactions.

#### 3.2.5 Thermal Conductivity Coefficient

The reference mixture without GHA additive (GHA0) has a relatively high thermal conductivity coefficient of 0.243 W/m-K (Figure 14). This value decreased systematically as the GHA content increased and reached a minimum value of 0.199 W/m-K in the GHA30 mix. This indicates that adding GHA can ameliorate foam concrete’s thermal insulation performance. The reduction in thermal conductivity corresponds to an approximate 18.1% decrement, which is commendable for nonstructural insulating materials. The major driving factor behind this decrement could be attributed to a diminished total unit volume weight of the matrix and an increased pore volume afforded by the GHA addition. From the earlier results, it can be recalled that fresh and dry densities decreased systematically with increasing GHA substitution rates. For example, the dry density for GHA0 was 1226 kg/m^3^, decreasing to 1085 kg/m^3^ in the GHA30 mixture. Thermal conductivity is directly related to a material’s density, where structures with higher densities conduct heat better through their solid phase. In contrast, more porous structures trap heat better to slow conduction. Thus, in this context, such a lightweight and highly porous structure, formed due to the GHA additive, decreased thermal conductivity by disrupting the heat conduction pathways.

**Figure 14:**
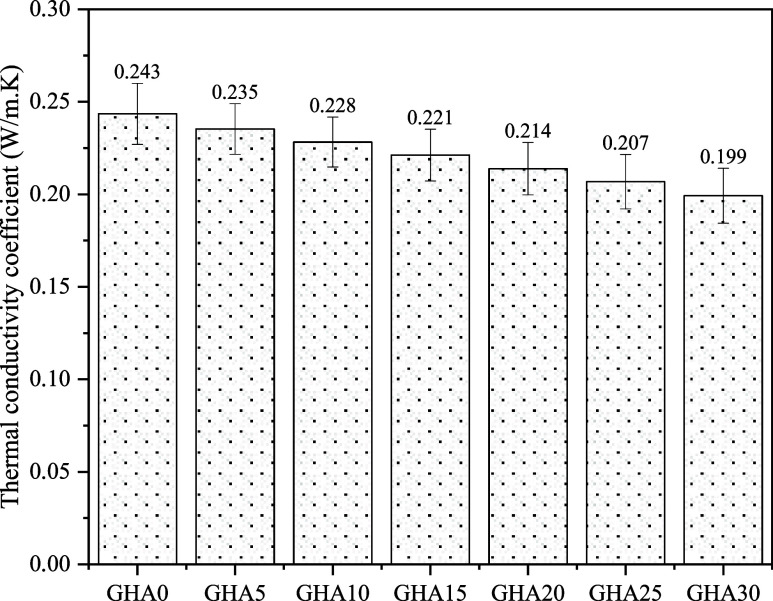
Thermal conductivity coefficient for samples.

This decline is also attributable to the physical properties and chemical structure of GHA. GHA has a delicate, fibrous, and amorphous ash form with low thermal conductivity. This physical structure creates microporous regions within the matrix that can be a thermal barrier. At the same time, the low density and high percentage of internal voids of GHA lead to a reduction of thermal bridges and interruption of heat flow within the concrete. This is another mechanism supporting the observed decrease in thermal conductivity. However, the high LOI (Loss on Ignition) value of GHA and the organic residues it contains also reduce the number of solid phases contributing to heat conduction. Organic structures generally have low thermal conductivity, which lowers the overall λ value. This effect is particularly pronounced at high substitution ratios, such as GHA25 and GHA30, and improves insulation performance. These results are consistent with other studies on agricultural waste admixed concrete in the literature. For example, it has been reported that admixtures such as POFA, RHA, and OWA similarly reduce thermal conductivity and provide better insulation performance. In this context, in addition to being a valuable resource in terms of sustainability, GHA stands out as a potential improver for foam concretes in terms of thermal insulation.

In the studies, adding agricultural waste ashes such as POFA further reduced heat transfer by creating air pockets in the lightweight concrete that act as heat insulators.^73^ Ashes such as POFA and fly ash enter into pozzolanic reactions, filling the voids and strengthening the concrete matrix, as a result of which the thermal conductivity is reported to decrease.^74^ The study by Eeydzah et al. shows that increasing POFA reduces the thermal conductivity in lightweight concrete due to its enhanced pore structure, leading to lower density and higher air voids, improving the insulation properties (0.48 W/mK at 30% POFA).^75^ In conclusion, agricultural waste ashes effectively reduce the thermal conductivity coefficients of lightweight or foam concrete. This effect is due to the porous structure, low density, and advanced pozzolanic reactions in the concrete mix. Including RHA reduced the foam concrete’s overall density, resulting in lower thermal conductivity. For example, a 12.29% reduction in thermal conductivity was observed with 10% RHA replacement.^76^ RHA has been reported to contribute to a more refined pore structure, increasing the air-trapping micropores within the matrix and thus improving the insulation properties.^77^ Similarly, garlic husk ash (GHA) used in this study systematically reduced foam concrete’s thermal conductivity coefficient, reaching a value of 0.199 W/m-K at a 30% replacement rate, a reduction of about 18.1% compared to the reference mix. This reduction aligns with the mechanisms reported in the literature for agricultural waste admixtures such as POFA and RHA. The low density, fine-grained structure and fibrous morphology of GHA promoted the formation of micro air voids within the matrix, creating an effective insulating barrier against heat transfer. At the same time, the late wet pozzolanic reactions developed due to the high silica content of GHA, which provided both binding properties and changed the shape and distribution of micropores, resulting in a more regular and insulating structure. In this way, not only were the heat conduction pathways interrupted, but the solid phase connections to conduct heat were also reduced. In the literature, POFA and RHA additives have been reported to reduce the thermal conductivity by reducing the density of the matrix; similarly, GHA improved the thermal performance by reducing the unit volume weight of foam concrete in this study. In this context, it is demonstrated that GHA is an effective mineral additive for reducing thermal conductivity, like other ashes of agricultural waste origin, and can produce sustainable building materials with high energy efficiency.

#### 3.2.6 High Temperature Resistance

In terms of high temperatures, the evaporation of free and bonded water, the decomposition of hydration products, the thermal degradation of certain minerals at high temperatures, and subsequently, weight loss are attributed to those high temperatures usually experienced after the combustion of cementitious materials. For example, the GHA0 reference mix experienced a weight loss of 7.56% at 250 °C, 15.50% at 500 °C, and 19.64% at 750 °C (as shown in Figure 15a). These values reflect the average response of foam concrete without GHA admixture to increasing temperatures. In contrast, the GHA admixtures displayed varying behavior at different temperatures. At 250 °C, the highest weight loss was observed in the GHA10 mix (22.32%). This is mainly because this mix has high porosity (32.12%) and high water absorption capacity (30.27%) as proven in previous experiments. These properties mean that more physically bound water is retained in the sample. Therefore, at low temperatures, the rapid evaporation of this water, which is not bound to the hydration products, can lead to high weight loss at an early age.

**Figure 15:**
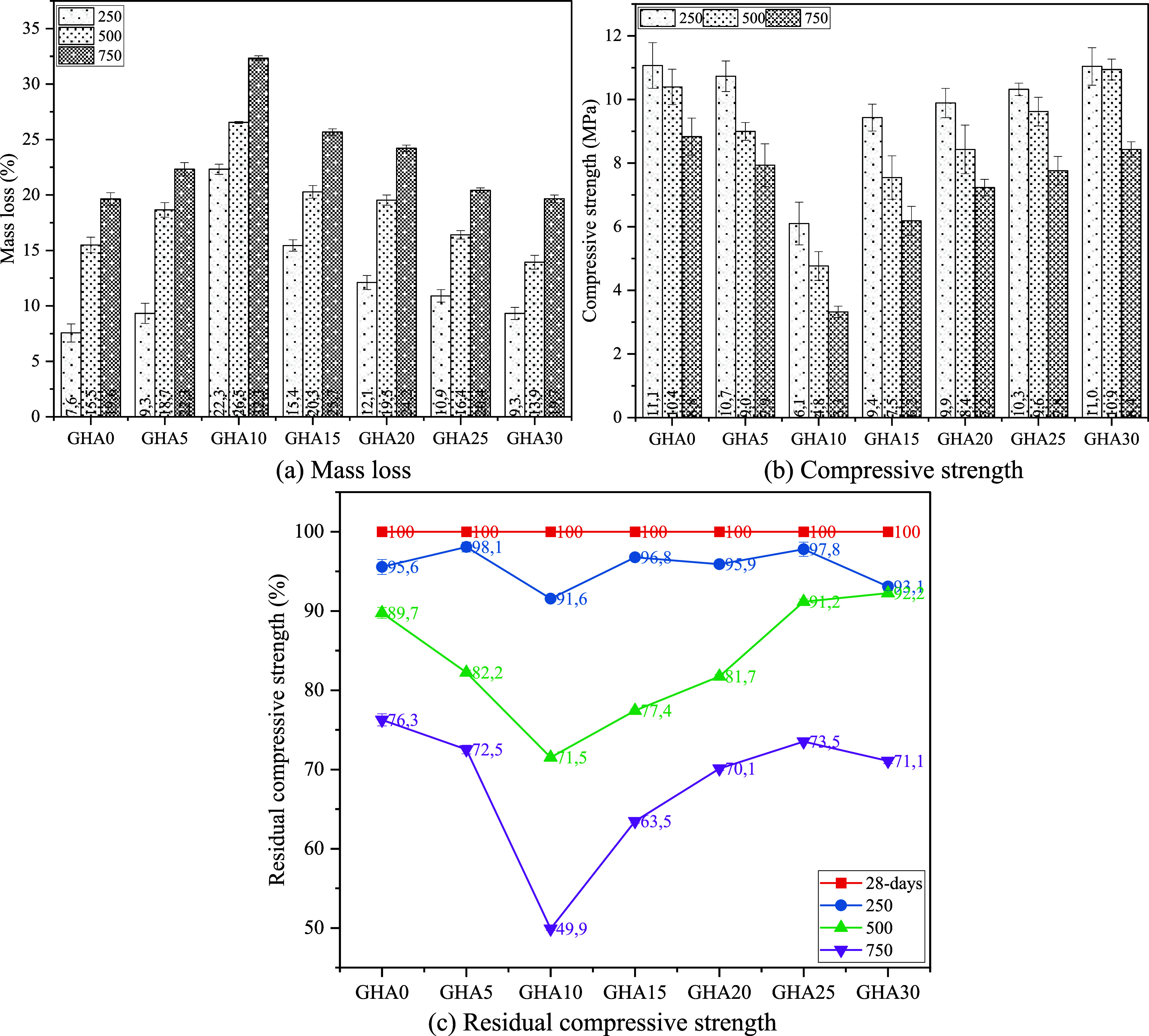
High temperature resistance of specimens.

Again, under the highest weight loss percentage of 26.54 and 32.34% for GHA10 at 500 and 750 °C, respectively. Processes such as decomposition of solid phases (C–S–H and Ca­(OH)_2_, for example), oxidation of organic residue, and pore structure collapse of matrix occur during this temperature range. A high LOI value of GHA (27.6%) indicates high chances of unburnt organic matter in this mixture, leading to further mass loss at temperatures of 500 °C and beyond. Simultaneously, high porosity facilitates vapor escape, accelerating mass loss. Conversely, blends with a high percentage of GHA, such as GHA30, demonstrate some of the best resistance to weight loss, with weight loss at 500 and 750 °C recorded at 13.94 and 19.66%, respectively, nearly equal to or lower than that of the reference mix. This improvement can be attributed to a dense, refined microstructure, low water demand, and reduced porosity resulting from the high GHA content at later ages. Additional C–S–H gels formed by GHA in the matrix through pozzolanic reactions contribute to a more heat-stable structure, limiting the formation of evaporable voids. This shows the increased thermal durability of these blends by maintaining mass stability at high temperatures. Also, lesser weight losses occurred in GHA5, GHA15, and GHA20 mixtures than in GHA10 in the temperature range between 500 and 750 °C. This may mean that above a certain percentage (especially after 15%), the pozzolanic effect of GHA takes over, closing up voids and stabilizing the internal structure of the matrix. This means that lower amounts of GHA enhanced the internal water holding capacity of the matrix, which, therefore, accelerated water loss and thermal degradation. Meanwhile, with higher amounts, GHA would enhance the temperature-resisting ability of the matrix by sealing those voids.

The weight loss of foam concrete under high-temperature conditions mainly depends upon parameters such as the amount of water present in the foam concrete, the porosity of the matrix, the organic matter content (LOI) of GHA, and microstructural density. Low-level GHA admixture (up to 10%) has adverse effects on these properties, while higher levels (25–30%) reverse these effects and create a more stable and heat-resistant structure. This shows that GHA can be an environmentally acceptable and strategic additive for thermal stability.

GHA 0, the reference mix without the GHA additive, achieved a compressive strength of 11. 58 MPa at 28 days. This mixture experienced a strength reduction of 4.4 to 11%. 07 MPa at 250 °C, 10.2 to 10%. 40 MPa at 500 °C, and 23.7% to 8.83 MPa at 750 °C (Figure 15b). GHA 0, the reference mix without GHA, achieved a 28-day compressive strength of 11.58 MPa. This mixture experienced a strength reduction of 4.4%, down to 11.07 MPa at 250 °C, 10.2%, down to 10.40 MPa at 500 °C, and 23.7%, down to 8.83 MPa at 750 °C (Figure 15b). These values illustrate the expected mechanical deterioration trend of conventional cement matrix foam concrete under the influence of temperature. Microstructural degradation of the binder phases with increasing temperature, particularly the segregation of C- S- H gels and calcium hydroxide crystals, was among the primary causes of strength loss. When analyzing the GHA admixtures, it is evident that their performance against heat differs significantly based on the admixture ratio. The GHA 10 mix, which had the lowest initial strength, showed only 6.66 MPa at 28 days. This reduced value was decreased to about 6.10 MPa at 250 °C by 8.4%, to 4.77 MPa at 500 °C by 28.4%, and to almost half its value, only 3.33 MPa at 750 °C. This deep attenuation is quite in consonance with earlier data on high porosity (32.12%), high water absorption (30.27%), and appreciable capillary water absorption. In turn, these structural features increased vapor pressure in the matrix with rising temperature, resulting in microcracking and collapsing the binder phases. In contrast, the GHA 30 blend exhibited the best temperature resistance. With a 28-day strength value of 11. 86 MPa, this mixture decreased to 11. 04 MPa at 250 °C, reflecting only a 6.9% loss, to 10.94 MPa at 500 °C, with a 7.7% loss, and to 8.43 MPa at 750 °C, showing a 28.9% decrease. These values indicate superior performance even compared to the reference mixture. The high-temperature resistance of GHA 30 can be attributed to the pozzolanic effects that promote microstructure condensation at a later age. Specifically, GHA in this mixture compensated for the voids caused by the decomposition of hydration products with more stable binder phases, forming a dense gel matrix and enhancing resistance to thermal degradation. Additionally, previously obtained data, such as low porosity (24.96%) and low weight loss (19.66%), confirm the excellent structural stability of GHA 30.

GHA15, GHA20, and GHA25 mixtures have moderate amounts of GHA additives and are therefore somewhat better than GHA10, but not as good as GHA30, when it comes to mechanical losses under high temperatures. For example, at 750 °C, the compressive strength of GHA25 dropped from 10.55 to 7.76 MPa, a reduction of approximately 26.5% from its 28-day compressive strength, which closely parallels the 23.7% reduction shown by the control mixture. This implies that GHA offers adequate structural support at around 25% but provides the best results against heat at an increased level of about 30%. Consequently, parameters that significantly influence high-temperature strength include the GHA admixture ratio, matrix density, porosity level, and stability of binder phases. Low GHA admixtures (like GHA10) compromise the structural integrity of foam concrete, while higher admixtures (GHA25-30) yield a denser and more heat-resistant matrix. These data clearly illustrate that GHA is a technical admixture assessed for its environmental sustainability, fire resistance, and high-temperature stability.

The results obtained in this study reveal a remarkable and scientifically significant relationship between capillary water absorption and high-temperature strength. Capillary water absorption is a critical parameter that reflects the water-carrying capacity of voids in the internal structure of concrete. Porous, capillary water-open structures exhibit poor resistance in terms of workability, water permeability, and high temperatures. Under the influence of elevated temperatures, the rapid evaporation of free water in these capillary spaces leads to increased internal pressure and cracking. This situation exacerbates temperature-induced strength losses, particularly in mixtures with strong pore connections.

Experimental findings support this mechanism. The GHA10 mix demonstrated the highest capillary water absorption value in the study (9.54 kg/m^2^) and the lowest compressive strength at 750 °C (3.33 MPa), indicating a loss of approximately 50%. In contrast, the GHA30 mix distinguished itself with the lowest capillary water absorption value (6.32 kg/m^2^) while exhibiting the best high-temperature performance, maintaining a compressive strength of 8.43 MPa at 750 °C with a loss of only 28.9%. This difference reveals the impact of capillary voids and joints on thermal stress distribution under high-temperature effects, suggesting that a less permeable microstructure can limit crack propagation by reducing evaporation-induced internal pressures. It is understood that the GHA additive enhances thermal stability and durability at high temperatures due to its regulatory effect on the capillary structure. This finding suggests that the capillary water absorption value should be considered concerning durability and as a predictor of high-temperature performance. Therefore, managing foam concretes’ capillary water absorption properties is crucial to enhance their usability under harsh environmental conditions, such as fire resistance.

Figure 15c illustrates the residual compressive strengths of foam concretes at various high temperatures. At 250 °C, all mixtures maintained a strength of over 90%. Since only physical water evaporation occurred at this temperature, the binder phase of the structure was not significantly damaged. However, GHA10 displayed a lower value of 91.6% compared to the others, indicating that this mixture is more brittle and porous in microstructure (high porosity and water absorption). The GHA30 blend showed remarkable stability even at this temperature, achieving a value of 93%. The 500 °C level is a critical threshold where C–S–H gels begin to decompose, Ca­(OH)_2_ undergoes thermal degradation, and microcracks become apparent. The graph illustrates that the effect of GHA additive ratios becomes more pronounced at this juncture. In particular, the GHA10 mixture exhibited the lowest residual strength at 71.5%. This signifies a strength loss of approximately 28.4% and demonstrates that the porous structure of GHA10 cannot withstand thermal shocks. Conversely, GHA25 and GHA30 blends exhibited the highest strength retention under high temperatures at 91.2 and 92.2%, respectively. The dense microstructure and low capillary absorption rates resulting from pozzolanic reactions that developed later in these mixtures account for this success. At 750 °C, a significant portion of cement-based systems begins to fail. At this temperature, the system’s physical and chemical integrity is compromised. The GHA10 mix maintained about 50% strength and showed the weakest performance. In contrast, the GHA30 mixture retained 71.1% strength even at this temperature, providing the best high-temperature stability. This value is slightly lower than the reference mix GHA0 (76.3%) but higher in absolute strength (8.43 MPa). GHA25 also stood out with 73.5%. Figure 15c clearly shows the effect of GHA admixture on the high-temperature performance of foam concretes. Optimum structural integrity and permanent strength results were obtained when the GHA admixture ratio increased to 25–30%. These results demonstrate that GHA is a technically sound and sustainable additive for creating fire-resistant construction materials.

Cementitious compounds from agricultural wastes like palm oil fuel ash (POFA) and rice husk ash (RHA) can significantly improve concrete’s high-temperature resistance. Rich in reactive silica, these materials interact with the calcium hydroxide in concrete to create a denser microstructure capable of enhancing thermal stability. The mechanism involves a pozzolanic reaction in which silica from the ash reacts with lime to produce additional C–S–H, thereby increasing strength and durability against elevated temperatures. This transition leads to a denser and less porous structure, critical for thermal resistance.^78–80^

The reactive silica in POFA and RHA reacts with calcium hydroxide to form C–S–H (thanks to pozzolanic activity), which is responsible for concrete’s strength and thermal resistance. This effect is reported to improve the high-temperature resistance of cement-based composites.^81^ These secondary C–S–H gels result in a denser microstructure, reduce porosity, and increase thermal stability.^82^ Ashes from agricultural waste, such as RHA and POFA, create a more open pore structure in concrete, facilitating moisture transport and reducing vapor pressure buildup during fire exposure. This property helps maintain concrete integrity under high temperatures.^83^ Adding these ashes also significantly reduces the thermal conductivity, with reductions of up to 42.68% reported in the literature. This insulating effect helps keep the internal temperature of the concrete lower during fire exposure.^84^ In this context, the GHA used in this study has similar pozzolanic potential and can form secondary C–S–H gels at late ages due to its high silica content. Experimental findings show that a denser, closed porous microstructure develops, especially at higher GHA ratios (25–30%), resulting in lower weight loss and higher residual compressive strength under high temperature. In addition, the 18.1% reduction observed in the thermal conductivity coefficient with GHA additive is in line with the insulation effect reported in the literature. It reveals that the material successfully provides internal temperature control. In this context, GHA stands out as an innovative mineral additive alternative that supports sustainability and fire-resistant building material production, exhibiting properties similar to those of known agricultural waste additives such as POFA and RHA.

Figure 16 shows the physical appearances of foam concrete samples with different GHA additive ratios exposed to temperatures of 200, 400, and 600 °C. There is no visible physical deterioration or color change in the samples exposed to 200 °C. The samples have preserved their integrity; no apparent damage, such as cracking, flaking, or discoloration, is observed on their surfaces. Since only free water evaporates at this temperature level, no separation or structural weakening has yet occurred in the binding phases. This is the temperature range where over 90% of the strength is preserved, as seen in the mechanical results obtained previously. When 400 °C is reached, it is noticeable that in some samples, surface color changes, slight graying, and especially in mixtures with low GHA content, minimal microcracks begin to form. This temperature level represents a critical threshold at which calcium hydroxide decomposition begins and microstructural deterioration first appears in the binder gels. The fact that mixtures with high GHA content (e.g., GHA25 and GHA30) appear relatively more compact and their integrity is preserved suggests that the developed microstructure in these samples absorbs thermal stresses more successfully. In the samples exposed to 600 °C, the color change became apparent, the surface tones turned from light gray to dark gray, and visible crack formations and surface flaking occurred in some samples. These deteriorations are observed more intensely in mixtures with low GHA content; on the other hand, samples with high content, such as GHA30, are in a better condition regarding structural integrity. This reinforces the idea that GHA limits the internal stresses caused by high temperature by densifying the microstructure and increasing the thermal stability of the concrete.

**Figure 16:**
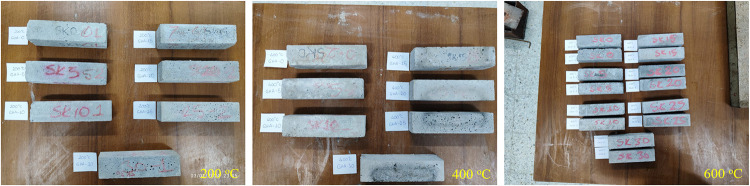
Physical appearance of samples after high temperature.

Figure 17 illustrates a visual analysis of the relationship between the 28-day compressive strength and the coefficient of thermal conductivity of GHA-blended foam concretes, with GHA content included as a third parameter. The upper left region of the graph showcases areas exhibiting both low thermal conductivity (approximately 0.199–0.205 W/mK) and high humidity at high pressure (11–12 MPa). This region also contains mixtures with the highest GHA additive rates (27–30%). Here, the pozzolanic activity of GHA demonstrates its ability to strengthen the mechanical bonds of the C–S–H gels, enhancing the durability of the matrices while preventing heat distribution gaps by forming a more closed and homogeneous microstructure. This microstructural benefit from the GHA additive supports load-bearing capabilities and positively impacts structural integrity. Moving toward the lower right section of the graph, the thermal conductivity coefficient rises to approximately 0.235–0.240 W/mK, while the compressive strength declines to 9 MPa and below. These areas correspond to mixtures with a low GHA additive ratio (0–6%). In agreement with earlier experimental data, these mixtures exhibit increased porosity, water absorption capacity, and a weaker binder phase. Such properties pose negative consequences, including increased heat conduction and decreased compressive strength, ultimately resulting in compromised performance for these mixtures. In the middle section of the graph, specifically within the thermal conductivity range of approximately 0.215–0.220 W/mK, transitional trends are noted for the compressive strength region of 10–11 MPa. This area depicts an equilibrium range where GHA contribution is around 15–20%, indicating that the microstructure is not yet fully optimized, but the performance level is acceptable. This range is particularly noteworthy as it illustrates that the effect of GHA, based on the contribution rate, is not linear and that pozzolanic effects become significantly active beyond a particular threshold value. Overall, this graph indicates that as the GHA additive ratio increases, foam concretes’ thermal insulation properties and compressive strength improve. The GHA additive facilitates a dual enhancement of foam concretes in terms of mechanical and thermal performance. Specifically, the 25–30% additive ratio highlights a threshold point where this performance peaks, marking it as the optimal additive level. In this regard, GHA, a sustainable resource derived from agricultural waste, can be considered an effective additive for producing heat-resistant and highly insulating building materials in foam concrete production.

**Figure 17:**
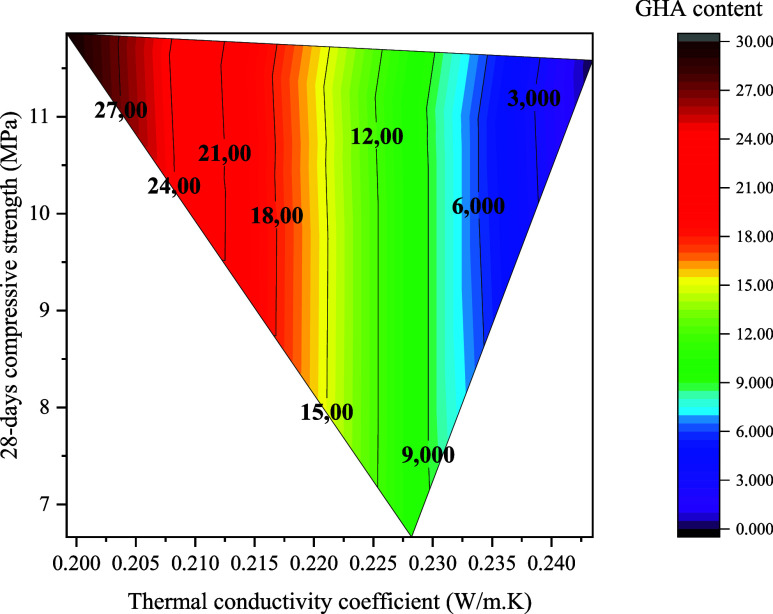
Relationship between 28-day compressive strength and thermal conductivity coefficient of GHA.

#### 3.2.7 Freeze–thaw Resistance

When Figure 18a is analyzed, it is seen that GHA substitution ratios significantly affect foam concrete’s durability. In particular, the GHA30 mixture showed the highest compressive strength of 10.32 MPa, but only experienced a weight loss of 3.17%. This indicates that the mix with high GHA content is highly resistant to freeze–thaw cycles. This success can be attributed to the dense formation of C–S–H gel in the microstructure of the matrix due to the high silica content of GHA. In addition, the microscopic structures contained in GHA limited the migration of capillary water by filling the voids, thus reducing the possibility of ice crystals causing volumetric expansion. This feature prevented water from freezing, expanding inside the matrix, and forming cracks. On the other hand, the GHA10 mixture showed the lowest strength (5.32 MPa) and the highest weight loss (5.91%). This situation caused the mechanical resistance to weaken when GHA was substituted below a particular threshold value due to the decrease in the amount of binder, and the matrix formed was not homogeneous enough. At the same time, the low calcium content of GHA at this substitution rate did not favor sufficient C–S–H formation, resulting in increased porosity and reduced resistance to freeze–thaw cycles. Generally, maintaining the GHA content in the 25–30% range preserved the mechanical strength and enhanced freeze–thaw resistance. The GHA30 mix exhibited approximately 2.7% greater compressive strength and 0.04% less weight loss than the reference mix, GHA0. This indicates that GHA is not only a sustainable additive but also a functional mineral additive that improves durability against environmental factors. In conclusion, this study demonstrates that using GHA positively impacts the freeze–thaw strength of foam concretes. Nevertheless, it is crucial to optimize the replacement ratio.

**Figure 18:**
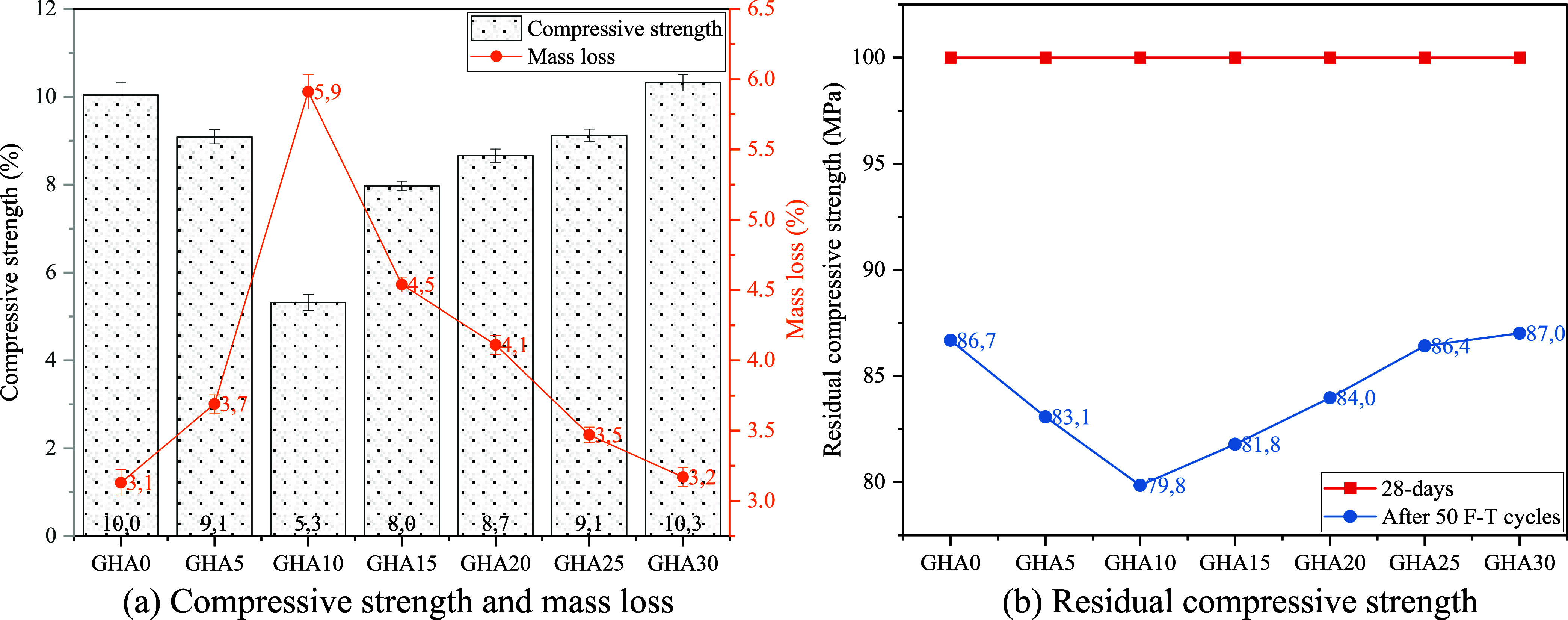
Resistance of the specimens to 50 cycles of F-T cycling.

Figure 18b shows the extent to which the residual compressive strength of the foam concrete specimens after 50 cycles of freeze–thaw (F-T) cycling is maintained in percentage terms compared to the 28-day reference values. GHA0 (reference mix) showed an excellent durability with a residual compressive strength of 86.7%. However, the most remarkable result belongs to the GHA30 mix, which retained the highest residual compressive strength of 87.0%. This indicates that the blend with a high proportion of GHA offers superior resistance to freeze–thaw cycles. That is, the mechanical strength was primarily maintained. This can be directly attributed to the microstructural effect of GHA. Regarding the impact of high silica GHA, it promotes C–S–H generation in a binder matrix, creating a denser, tighter, and less permeable structure that limits water penetration and freezing-thaw expansion by preventing internal damage. Conversely, GHA10, with the least strength ratio at 79.8%, is the mixture worst affected by the freeze–thaw effect. This can be explained by the decrease in the binder and the insufficiently developed C–S–H structure at low GHA content. Insufficient densification of the structure and high porosity resulted in easier penetration of water and increased risk of cracking. GHA20 and GHA25 mixtures succeeded with 84.0 and 86.4% residual strengths, respectively. This indicates that 20–30% GHA substitution may be the optimum range for the freeze–thaw strength of foam concrete. These findings support the high potential of GHA in developing long-lasting, environmentally friendly building materials.

RHA increases the compressive strength up to a specific limit (20% substitution), but after this point, the F-T resistance decreases due to increased porosity. RHA’s high specific surface area improves the pore structure by providing hydration nucleation sites and improving the interfacial transition zone (ITZ). The porous structure of RHA can lead to significant water absorption during freeze–thaw cycles, causing internal pressure and potential damage.^85^ Tambichik et al. reported that the F-T resistance of concretes can be increased by using POFA and RHA in an optimum ratio.^81^ The effect of agricultural waste ashes on freeze–thaw resistance has been reported to depend on the balance between a denser microstructure resulting from pozzolanic reactions and increased porosity.^86,87^ Hong et al. reported that the freeze–thaw resistance of PCM concretes with RHA content increased.^88^ Jin et al. reported that the freeze–thaw resistance of self-compacting concretes produced using 30% biomass fly ash improved.^89^ Based on this literature review, the findings of your study on the freeze–thaw (F-T) strength of GHA (garlic husk ash) admixed foam concretes show a significant parallelism with the high residual compressive strengths and low weight losses obtained, especially at 25–30% GHA substitution rates. In the literature, it has been reported that F-T resistance can be increased with the optimum use of agricultural waste ashes such as RHA and POFA, and this is based on factors such as dense microstructure formation, an increase in hydration products, and control of micro voids. Accordingly, our study shows that GHA has a similar potential to improve F-T strength at the optimum usage range (25–30%). Particularly with a high percentage of GHA substitution, secondary C–S–H gels formed through pozzolanic reactions made the matrix more compact, minimizing water penetration during freezing and preventing microcrack formation due to internal expansion. It is understood that GHA has a similar microstructure, with a densifying and ITZ improving effect due to its high specific surface area and active silica content. In this context, our study also shows that GHA has an essential feature in enhancing the freeze–thaw resistance of foam concretes.

### 3.3 Microstructural Investigations

#### 3.3.1 Microstructure of Samples after 90 Days of Curing

The microstructure of the GHA0 mixture cured for 90 days is shown in Figure 19a. In the SEM image on the left, the ‘clustered C–S–H gels’ (agglomerated calcium silicate hydrate gels) are very prominent. These gels are the most crucial binder phase resulting from cement hydration and are the primary source of mechanical strength in foam concrete. The image reveals that these gels are clustered in a fibrous and dense structure. This fibrous structure represents the typical morphology of C–S–H gels, contributing to increased mechanical strength, particularly at later ages (90 days). The density and distribution of C–S–H in the image suggest that the hydration of the sample has advanced significantly, indicating favorable water-cement reactions. Portlandite (Ca­(OH)_2_, CH) phases, identifiable as substantial, slab-like crystals, are observed in the SEM image on the right. Such crystals will be caused by hydration and indicate excess calcium in the microstructure. Although Portlandite phases do not add to mechanical strength, they generally reduce it and limit durability because they tend to increase porosity. The size and density of the Portlandite crystals in the image indicate that the sample lacks pozzolanic activity, meaning this phase remains unconverted without a pozzolanic additive such as GHA. These observations align with the experimental data for the GHA0 specimen, which shows a lower late age strength increase, higher porosity, greater water absorption capacity, and relatively lower durability. Without the GHA admixture, the Portlandite phase formed due to cement hydration, which was more prominently observed in the microstructure since it did not react with a reactive silica source to yield additional C–S–H.

**Figure 19:**
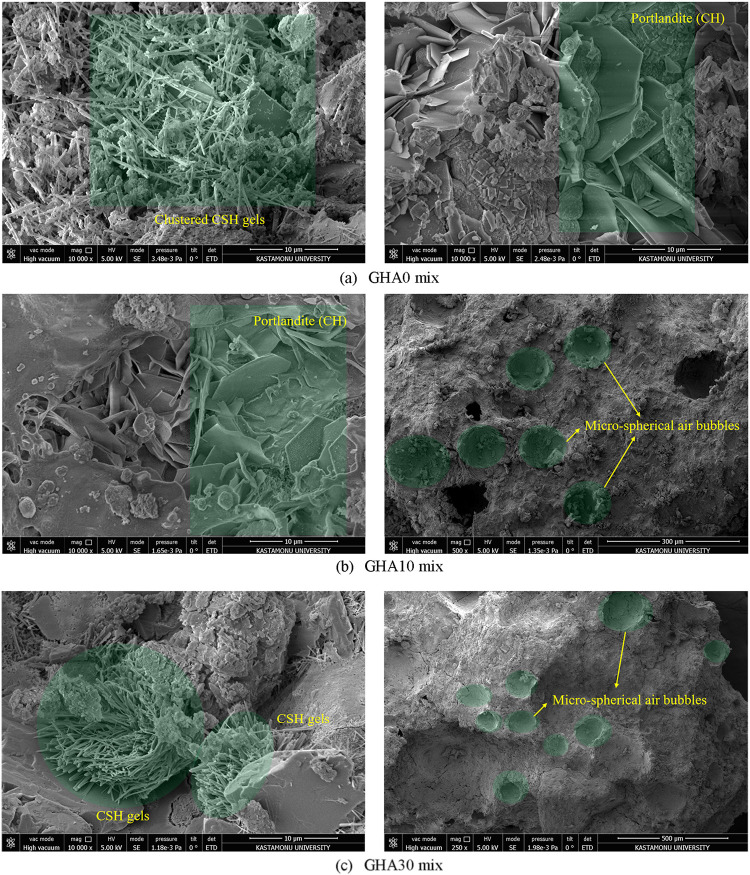
Microstructures of 90-day cured specimens.

Figure 19b presents the microstructure investigations of the GHA10 mixture. In the 10,000× magnification image on the left, Portlandite crystals (Ca­(OH)_2_, CH) with a slab-like morphology are visible. The presence of these crystals signifies that a certain amount of CH, a hydration product, remains in the system despite the GHA contribution. However, it is noteworthy that Portlandite crystals are less dense and more fragmented than those in the GHA0 sample. This indicates that CH is partially consumed and transformed into C–S–H gel due to the pozzolanic activity of the GHA additive. This structure aligns with the experimental results, where GHA10 exhibited poor mechanical properties, including lower compressive strength, higher porosity, and better water absorption. This occurs because the GHA ratio cannot consume CH entirely at this level. Given that unreactive or low-reactive phases remain in the microstructure, C–S–H production is limited, resulting in poor binding. In the 500× magnification image on the right, the microspherical air voids characteristic of foam concrete are visible. These voids are integrated into the structure thanks to the foaming agent used and contribute to the lightness of the concrete. In the GHA10 specimen, the size and distribution of these voids appear pretty heterogeneous. The nonhomogeneous distribution of air voids, their accumulation in certain areas, and the interconnectedness of some pores have caused this specimen to display characteristics such as high porosity, elevated capillary water absorption, and lower density. This situation is also directly related to the matrix’s inability to compact adequately due to the insufficient GHA additive ratio.

SEM images of the GHA30 mixture cured for 90 days are presented in Figure 19c. The most important structure in this 10,000× magnification image is the dense, needle-like, and fiber-like C–S–H (calcium silicate hydrate) gels. The majority of these gels are formed by the pozzolanic reaction of reactive silica (SiO_2_), alumina (Al_2_O_3_), and Portlandite (Ca­(OH)_2_) phase in GHA. Thanks to the exceptionally high surface area and amorphous structure of GHA, these pozzolanic reactions occurred faster and more efficiently, resulting in a dense gel matrix. C–S–H gels are the main binding phase of the cement paste, and the dispersed agglomerated structures in the image show secondary gel formations that favor strength development at late ages. This explains why specimen GHA30 achieved higher values of 90-day compressive strength than GHA0. In this 500× magnification image, microscopic air bubbles are visible. These bubbles form the low-density and insulating structure characteristic of foam concrete. However, what is important here is that these air pockets are smaller in diameter and homogeneously distributed. With increasing GHA admixture, the cement paste becomes more viscous, which leads to a more even distribution of the foams in the mix. The more uniform air bubble distribution interrupts the heat conduction pathways and contributes to the low coefficient of thermal conductivity (0.199 W/mK for GHA30). At the same time, since these voids are microscopic, they reduce capillary water transport and porosity, which is supported by low water absorption and porosity values. The SEM image of the GHA30 specimen clearly shows that the agricultural waste-based GHA admixture effectively consumed the Portlandite phase by pozzolanic reactions and formed secondary C–S–H gels in its place. This resulted in a microstructure that increased strength, reduced porosity, and improved thermal insulation. Furthermore, the homogeneous distribution of the foams shows that the blend is also optimized in terms of workability and foam stability.

#### 3.3.2 Microstructure of Specimens Exposed to High Temperature (600 °C)

The SEM microstructure analysis of GHA0 blend samples heat-treated at temperatures up to 600 °C is displayed in Figure 20a. In the 1000× magnification image on the left, the most predominant damage is microcracked, termed “map-like cracks.” This crack type is associated with the destruction of C–S–H gels at high temperature, especially during the change from Portlandite (Ca­(OH)_2_) to calcium oxide (CaO) by dehydration above 400 °C for such transformations. This produces volumetric shrinkage and internal stresses that lead to crack formation. These cracks show a regular, interconnected network structure, indicating that the thermal shock effect produces a continuous microstructural degradation of the foam concrete matrix. This confirms that more than 20% of strength loss was observed at 600 °C at the microscopic level. This 500× magnification image shows the structural deterioration on a more significant surface section of the GHA0 specimen. The cracks spread on the surface, around the pores, and formed a dense crack network in the matrix. Since no additional pozolanic additive exists in the GHA0 mixture, most of the portlandite phase remains unconverted, and secondary C–S–H gels are not formed to resist cracking at high temperature. This leads to poor performance in terms of both strength and microstructural integrity. At 600 °C, the compressive strength of the GHA0 specimen decreased by 23.7% (11.58 → 8.83 MPa). The total weight loss at this temperature was also 19.64%. The thermally induced cracks increased the void volume, favoring density loss and increased capillary water absorption. The microstructural deterioration observed in the GHA0 specimen exposed to 600 °C reflects the destructive effect of high temperature on the binder phases. Map-like cracks and a dense network are the main reasons for weakening concrete’s mechanical performance.

**Figure 20:**
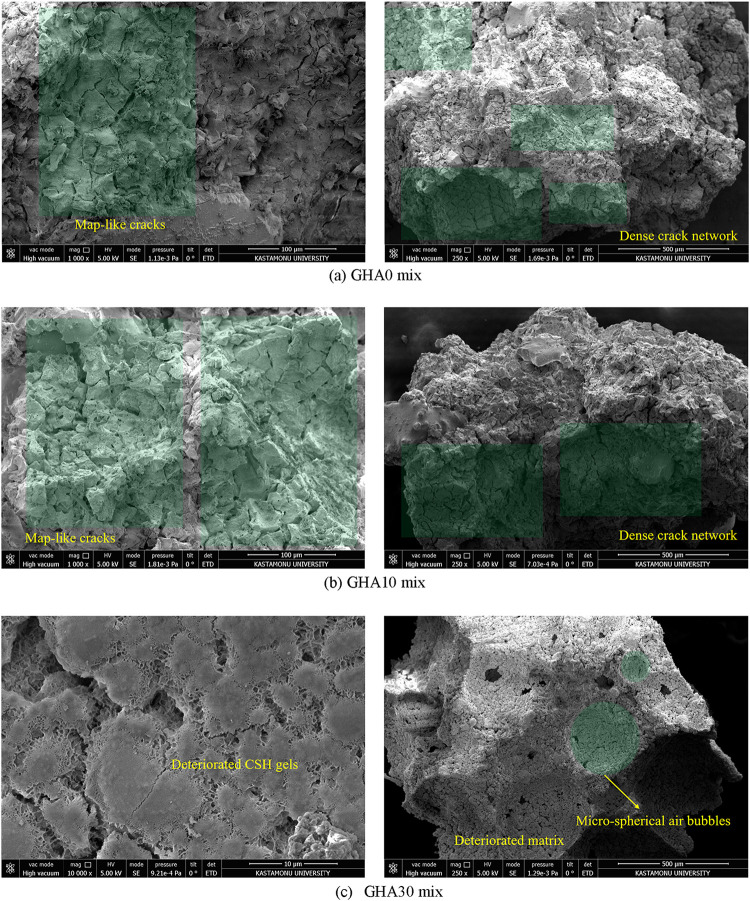
Microstructure images of 600 °C exposed specimens.

SEM images of the GHA10 mixture exposed to 600 °C are presented in Figure 20b. The irregular cracks, identified as “map-like cracks” in the left image, are classic signs of thermal damage due to high temperature. The cracks are associated with the onset of degradation of C–S–H gels at 600 °C, Portlandite Ca­(OH)_2_ decomposition, and calcium silicate phase recrystallization. What stands out is that the crack distribution in sample GHA10 seems more irregular and less controllable than in GHA0. This indicates that the reactive silica contained in GHA makes the cement paste matrix more resistant by forming additional C–S–H gels at high temperatures. In addition, the fine particle structure of GHA partially limited crack propagation by better filling the voids. The 250× magnification image on the right side shows a dense crack network structure. However, these cracks are less widespread and have a more limited depth than the GHA0 sample. Especially in the cement matrix, it is seen that the cracks are clustered in certain regions, but not wholly dispersed. This shows that the GHA admixture supports the pozzolanic reactions that provide heat resistance. This image signifies a more homogeneous matrix formation in the microstructure. This structural order contributes to better distribution of stresses due to thermal expansion and control of internal pressure accumulations that may occur due to evaporation. This is an effect that reduces crack propagation. The GHA10 specimen experienced a 50.0% strength loss up to 600 °C (6.66 MPa → 3.33 MPa). This is a higher rate than the GHA0 sample. However, fresh and hardened state properties such as increased porosity (32.12%) and high water absorption (30.27%) indicate that this mixture has a weaker internal structure. In other words, these microstructure images confirm the reasons for the strength reduction, but also reveal that the crack distribution and structural deterioration are more regular than in GHA0. The microstructure of the GHA10 mix after 600 °C reflects a combination of both thermal damage and the limiting effect of GHA. Secondary C–S–H gels formed by the pozzolanic effect of GHA provided partial resistance to crack formation; however, due to the initial porosity level of the mixture and workability problems, the strength decreased significantly.

Figure 20c shows the SEM images of the GHA30 mixture exposed to 600 °C. The left image shows that the compact, needle-like structure of C–S–H (calcium silicate hydrate) gels has been disrupted and transformed into an irregular and segregated form. Such morphological change occurs due to water loss and recrystallization in the internal structure of C–S–H gels, especially at temperatures such as 600 °C. At high substitution ratios such as GHA30, the initially formed C–S–H gels may already be more weakly bonded, so the disintegration process of these gels may be accelerated with temperature. In addition, this image clearly shows that the microstructure loses its homogeneity and the gel phase disintegrates and weakens the carrier skeleton. This situation also microstructurally supports the decrease in compressive strength observed after 600 °C. However, CSH gels must maintain their presence at 600 °C. Different reasons could explain this. When the chemical composition of GHA is analyzed, it is seen that it contains approximately 25.8% SiO_2_. This reactive silica reacts with Ca­(OH)_2_ during the cement hydration to form secondary C–S–H gels. These gels are generally more compact, denser, and may degrade later than conventional cement gels. In this context, it is thought that most of the gels formed in the GHA30 mix are these secondary C–S–H gels; therefore, their thermal strength is higher. The high GHA content reduced the ability of the matrix to conduct heat. The coefficient of thermal conductivity is already as low as only 0.199 W/m-K for GHA30. This delays or limits the direct exposure of the microstructure in the internal regions to this temperature, even if the outdoor temperature is 600 °C. This allowed the C–S–H gels, especially in the inner part of the sample, to be less affected by heat and to maintain their structure. The microspherical air bubbles in the SEM images formed a barrier against heat. Thanks to these voids, heat conduction was slowed down, which caused the temperature exposed to the gels to drop below the critical degradation temperature. Additionally, the air voids helped dissipate the vapor pressure and reduce gel disintegration.

In the image on the right, the degraded matrix structure is visible. Microspherical air voids, especially common in foam concretes, have become prominent. These voids have reached even sharper and more pronounced boundaries due to the separation of the surrounding gels and binder phases under the influence of high temperature. In addition, the homogeneity of the matrix was severely impaired, the structure loosened, and the connections were broken. The initially high porosity (24.96%) and low density (1121 kg/m^3^) of the GHA30 mixture caused the integrity of this microstructure to dissolve more easily with temperature. This is microscopic evidence of the high strength loss (about 29% decrease). SEM analysis of GHA30 mixtures exposed to 600 °C reveals the limits of the capacity of high GHA addition to provide heat resistance. Low-density structures with more air voids initially cause the matrix to lose its cohesiveness with temperature. It leads to dissolution of the C–S–H gels, an increasing number of cracks, and collapse of the load-bearing framework. Reactive silica in the GHA content is typically expected to provide a pozzolanic contribution; however, an elevated ratio of up to 30% can compromise microstructural integrity, particularly at critical temperatures such as 600 °C. Thus, it should be an optimum amount of the GHA contribution that can limit the actual heat resistance.

## 4 Conclusions

This study evaluated garlic husk ash (GHA) as a binder material in foam concrete production. The effects of GHA on fresh-state properties, mechanical strengths, microstructure, thermal conductivity and durability behaviors were investigated in detail. The experiments showed that GHA caused significant changes in the performance of foam concrete depending on the substitution rate. Approximately 30% of the garlic husks produced worldwide are released from approximately 20 million tons of garlic. 10% of the released garlic husks are also obtained from burning. In other words, approximately 600,000 tons of GHA can be obtained annually by burning these garlic husks. This amount is close to the cement consumption of developing (medium-sized) countries. More GHA can be released with the garlic consumption, which will increase in parallel with population growth. Therefore, using GHA as a cement substitute will be essential in reducing carbon emissions.

In this study, GHA, an agricultural waste, was evaluated as a partial replacement for cement in foam concrete production. The findings revealed that the use of GHA significantly impacted the mechanical, physical, and durability properties of the mixtures. Particularly, 25–30% replacement rates stood out in terms of compressive strength and durability, with the GHA30 mixture achieving a compressive strength of approximately 11 MPa, outperforming the reference mixture. The thermal conductivity coefficient of the same mix decreased to 0.199 W/mK, providing a significant advantage in terms of energy efficiency.

Durability tests support these results. After freeze–thaw cycles, GHA-added mixes mostly kept their strength; the GHA30 mix performed best, maintaining up to 87% of its compressive strength. The microstructure became denser, pores were partially filled, and there was little strength loss due to GHA’s delayed pozzolanic reactions at high temperatures. This shows that GHA enhances both long-term durability and early age performance.

All things considered, adding garlic husk ash to foam concrete improves its mechanical and thermal properties as well as its environmental sustainability. By utilizing agricultural waste, waste management problems are reduced and low-density, energy-efficient, and environmentally friendly building materials can be created. In this context, the use of GHA in foam concrete manufacturing opens up exciting new research opportunities.
